# Modelling Net CO_2_ Assimilation of Two *Sphagnum* Species From Temperature and Water Content Response

**DOI:** 10.1111/ppl.70325

**Published:** 2025-06-13

**Authors:** Alicia V. Perera‐Castro, Miquel Nadal

**Affiliations:** ^1^ Department of Botany, Ecology and Plant Physiology Universidad de La Laguna La Laguna Canary Islands Spain; ^2^ Institute of Biology University of Hohenheim Stuttgart Germany; ^3^ AgroParisTech, INRAE, UMR Silva Université de Lorraine Nancy France; ^4^ Departamento de Sistemas Agrícolas, Forestales y Medio Ambiente Centro de Investigación y Tecnología Agroalimentaria de Aragón (CITA) Zaragoza Spain

**Keywords:** carbon balance, photoperiod, primary productivity, respiration, *Sphagnum*

## Abstract

Photosynthesis and respiration respond differently to the combined effects of temperature and water status. Quantifying their responses is crucial to predict the carbon balance of *Sphagnum* peatlands in different scenarios of climate change. A first approach was done for two *Sphagnum* species inhabiting a boreal peatland in Finland. Gas exchange at different temperatures and moss hydration were measured to model net assimilation using simultaneous measurements of photosynthesis and dark respiration. In addition, measurements of moss surface temperature at different water content were performed in the field, covering natural conditions of sun exposure and air temperature. We also accounted for the interaction effect between moss canopy temperature and air temperature, radiation, and water content. Our model accurately predicted net assimilation and was used to estimate net primary productivity based on meteorological inputs and moss water content. The two *Sphagnum* species presented optimum temperatures for net CO_2_ assimilation around 25°C, with minimum changes at other temperatures. In contrast, dark respiration increased exponentially with temperature, which makes losses of carbon during the night and the duration of dark conditions key determinants in the carbon balance of *Sphagnum*. The modeled net primary productivity revealed an enhancement of CO_2_ fixation under warming conditions (averaged +10°C), concomitant to the expected transformation of peatlands from sink to source of CO_2_. Our model highlighted the importance of respiration restriction in ensuring positive assimilation in *Sphagnum*. Therefore, day and night temperature oscillation and short night photoperiods are more important than the optimum temperature of photosynthesis for carbon balance.

## 1 Introduction

Boreal peatlands are valuable ecosystems dominated by the moss *Sphagnum*, characterized by a low decomposition rate of dead plant material that results in the accumulation of 20%–30% of the global terrestrial carbon, despite occupying only a 3% of the Earth's surface (Vile et al. 2011). The net carbon budget in peatlands depends on the balance between two processes: (1) photosynthesis‐driven CO_2_ fixation and (2) decomposition‐driven CO_2_ and CH_4_ emissions (Limpens et al. 2008). Thanks to cool temperatures and waterlogged conditions that decrease oxygen availability, the decomposition rates are normally reduced, and carbon is accumulated in the soil. However, changes in temperature and hydrological processes under climate change may drive enhanced decomposition and changes in fungal communities, favoring the activity of decomposers (Dieleman et al. 2016; Asemaninejad et al. 2018; but see also Flanagan and Syed 2011; Nakanishi and Tsuyuzaki 2024). These new conditions could lead peatlands to behave as net carbon sources instead of sinks, depending on the new balance between carbon gain and decomposition rates. In addition to studying the drivers of decomposition rates, many efforts have been made in modeling past and future net ecosystem productivity in peatlands (Wu and Roulet 2014; Beyer and Höper 2015; Lunt et al. 2019; Chaudhary et al. 2020; Qiu et al. 2020; Zhao and Zhuang 2023) and establishing a relationship between ecosystem photosynthesis, respiration, and/or net ecosystem production with variation in temperature, water table depth, and radiation in temperate and tropical peatlands (Flanagan and Syed 2011; Charman et al. 2013; Kurnianto et al. 2015; Walker et al. 2017; Karki et al. 2019; Bengtsson et al. 2021; Hanson et al. 2020). However, the specific response of CO_2_ assimilation to changing temperature conditions and to the water content of the moss tissue remains underexplored in *Sphagnum*. Precise knowledge of how temperature, radiation, and water content‐and their interplay‐affect both photosynthesis and respiration is required to accurately predict the response of net CO_2_ assimilation and hence provide a more detailed understanding of the dynamics of carbon balance in peatlands.

Gametophytes of mosses are poikilohydric organisms, that is, their water content is equilibrated with atmospheric water potential (Walter 1931). The absence of stomata and, in most cases, the absence of a hydrophobic cuticle and endohydric conducting apparatus contribute to this poikilohydry (Raven 2002; Proctor and Tuba 2002; Raven and Edwards 2004; Vitt et al. 2014; Duckett and Pressel 2022). Delayed dehydration can only be achieved in some species thanks to a set of tiny, compact shoots that confer canopies the ability to retain high amounts of external capillary water and thick boundary layers (Dilks and Proctor 1979; Proctor et al. 1998; Proctor et al. 2007). This capacity to buffer water availability in the environment is most prominent in species with hyaline cells (organelle‐free, porous cells), including *Sphagnum* (Thompson and Waddington 2008). Mosses are frequently considered inherently better adapted to cold conditions than angiosperms (Glime 2007). Furness and Grime (1982a) and Furness and Grime (1982b) reported an average optimum temperature for the relative growth rate of 19°C, with long‐term temperatures of 30°C being lethal for all studied mosses. However, these previous studies confound the processes of photosynthesis and respiration. A comprehensive review of optimum temperatures for maximum net carbon assimilation in mosses recently found that most species have optimal temperatures around 25°C–30°C (temperature at moss surface) with no significant latitude effect (Perera‐Castro, González‐Rodríguez, and Fernández‐Marín 2022). Notably, species which grow in environments where the average maximum air temperature does not exceed 5°C (for instance, mosses inhabiting Antarctica) do not significantly differ from the 25°C–30°C optimum. This discrepancy between the growth and assimilation rates at elevated temperatures may have a methodological explanation related to the experimental procedures used for growing mosses under controlled conditions. Furness and Grime (1982a) and Furness and Grime (1982b) used an unrealistic photoperiod of 12 h and no thermal oscillation between day and night, as in some other studies culturing mosses (Bates 1994; Duckett et al. 2004; Melosik and Såstad 2005; Cove et al. 2009; Graham et al. 2010), being a 12/12 h photoperiod less frequent (Grime et al. 1990; Gerdol and Vicentini 2011; Horsley et al. 2011). This would result in excessively high respiration rates at high temperatures, thus lowering the capacity for carbon assimilation (Perera‐Castro, González‐Rodríguez, and Fernández‐Marín 2022). The reduction of night respiration by shortening night length or by experiencing low night temperatures could be crucial for positive carbon balance in mosses. For instance, moss survival in Antarctica may be more related to their reduced respiration at low temperatures during short nights instead of having lower optimum temperatures for photosynthesis (Perera‐Castro et al. 2020; Yin et al. 2023). The response of moss respiration to rising temperatures is relevant for modelling the effect of different climate change scenarios on the long‐term net CO_2_ assimilation and carbon balance of mosses, including *Sphagnum* species, separately from the response of peat decomposition and heterotrophic CO_2_ and CH_4_ release.

Water availability and temperature are the most relevant indicators of *Sphagnum* distribution (Campbell et al. 2021). However, a wide range of optimum temperatures for net CO_2_ assimilation has been reported for *Sphagnum* species in boreal ecosystems, from 6.5°C to 30°C (Stålfelt 1937; Rudolph 1968; Grace and Marks 1978; Silvola and Heikkinen 1979; Silvola and Hanski 1979; Titus and Wagner 1984; Lösch et al. 1994; Oechel and Van Cleve 1986; Gaberščik and Martinčič 1987; Goulden and Crill 1997; Bergeron et al. 2009), although in most studies the response of net CO_2_ assimilation to temperature was measured repeatedly using the same samples with no control of water content. For instance, this is the case for the only report (to the best of our knowledge) of the optimum temperature for *Sphagnum* in Finland 43 years ago (Silvola and Heikkinen 1979). By using air temperature instead of moss surface temperature, the authors predicted that an increase of 2°C would result in a null net change in the carbon content of the peatland (Silvola and Hanski 1979). However, ecologists have recently pointed out the importance of a detailed description of microclimate in understanding and modelling ecosystem functioning, especially in small‐stature species (Convey et al. 2018; Lembrechts et al. 2019; Lembrechts and Lenoir 2020). Surface temperature is especially dependent on incident irradiance, so mosses can experience high temperatures even in Antarctic ecosystems where air temperature can be 10°C–27°C lower (Matsuda 1968; Schenker and Block 1986; Smith 1996; Perera‐Castro et al. 2020). Therefore, a subsequent decrease in cloud cover (Mendoza et al. 2021) may have a warming effect on mosses. In addition, the water content of the live tissue also influences the heating of mosses so that excess water also buffers against extreme temperatures due to the high specific heat capacity of water (Pannewitz et al. 2005; Block et al. 2009; Perera‐Castro et al. 2020). The effect of the water content has scarcely been considered in the temperature response of CO_2_ assimilation in mosses, despite its critical role in the response of photosynthesis to air temperature and vapour pressure deficit in vascular plants (Li et al. 2020; Márquez and Busch 2024). In this sense, we hypothesize that the effects of high air temperature and high irradiance on photosynthesis and respiration will be exacerbated under lower water contents, resulting in a potential synergic effect of the temperature and water content, further limiting net assimilation under these ‘dry conditions’. In our study, we also consider the irradiance and water effects on the *Sphagnum* surface to estimate the present and future carbon balance of *Sphagnum*. Hence, we aim to estimate the carbon balance of *Sphagnum* by combining three driving factors and their interactions: temperature, irradiance and water content. Using this combined approach, we provide a much more accurate description of the temperature effect on *Sphagnum* carbon balance, including the environmental requirements (night and day air temperature, irradiance regime, etc.) that enhance net primary productivity in the ecosystem. Although applied to Finland peatlands, this new approach for assessing environmental responses can be readily extensible to other *Sphagnum* communities worldwide.

The objectives of the present study were three‐fold: (1) to quantify the response of respiration and photosynthesis of *Sphagnum* species to independent changes in temperature and water content, (2) to measure the effect of water content on the warm‐up of irradiated *Sphagnum* at the surface level in comparison with air temperature, and (3) to estimate the carbon balance of *Sphagnum* under different scenarios of temperature, water content, radiation, and photoperiod, considering optimum and suboptimum conditions for CO_2_ assimilation and the combined effects of these factors. All measurements were conducted in situ (Hyytiälä, Finland) with *Sphagnum* shoots collected during the growing season (3rd–17th July). While the measurements were conducted during a relatively short period in the peak growing season, this timeframe coincides with the period of highest physiological activity for *Sphagnum*. As such, our data capture the species' photosynthetic response under optimal conditions. Nonetheless, we acknowledge that *Sphagnum*‐dominated peatlands can remain active year‐round and thus experience different environmental conditions from those evaluated in the present study. Although our results do not necessarily provide a complete annual picture of carbon dynamics, they are valuable representatives of peak‐season dynamics.

## 2 Materials and Methods

### 2.1 Study Site and Plant Material

**FIGURE 1:**
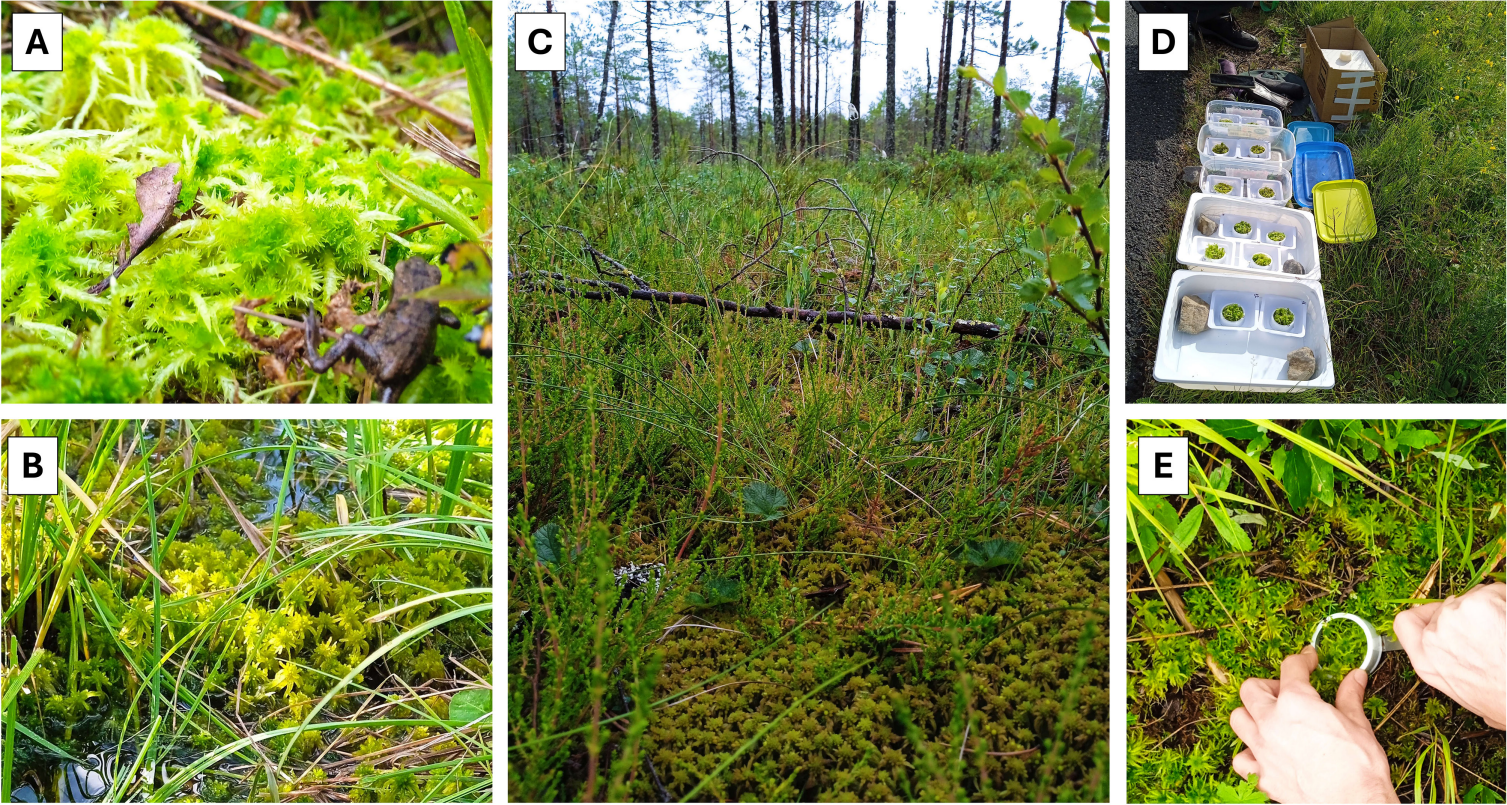
Study site and measured species. (A) *Sphagnum squarrosum* , (B) *Sphagnum angustifolium* . (C) General aspect of the peatland where mosses were collected for gas exchange analysis. (D) Set for in situ measurement of the effect on moss temperature of water content and irradiance. (E) Extraction of moss canopy in its original structure.

Two *Sphagnum* species were considered in this study: *Sphagnum squarrosum* Crome (Figure 1A) and *Sphagnum angustifolium* (Russow) C.E.O. Jensen (Figure 1B), growing at the fen border near Lake Kuivajärvi (61°50′ 21.7″ N, 24°17′13″ E), close to Hyytiälä Forestry Research Station (SMEAR II, Finland) (Figure 1C). For gas exchange analysis, apical capitula were daily collected and stored in semi‐sealed bags until their measurement within the next 4 h at the SMEAR II lab facilities. Adjacent samples were used for in situ measurements of moss temperature, radiation, and water content (Figure 1D,E). The collection dates comprised from 3rd July to 17th July 2023, from 9:00 to 10:00 h (local time). During this period, air temperatures oscillated from 20°C–25°C during the day to 10°C–15°C during the night, and maximum radiation was 500–800 W m^−2^ during the midday (SMEAR II meteorological station). In all cases, samples were collected randomly without considering their position with respect to hummocks or hollows and therefore their distance to the water table.

### 2.2 Gas Exchange Analysis

Measurements of net CO_2_ assimilation rate (*A*_N_), dark respiration rate (*R*_D_) and chlorophyll fluorescence in response to variations in water content (‘dehydration’ or ‘water loss’ curve; Perera‐Castro et al. 2020) at different temperatures were done with a Li‐6800 system (Multiphase Flash Fluorometer chamber, LiCOR Biosciences). Each dehydration curve was performed in samples that were oversaturated with distilled water prior to the measurements; then, they were placed over a custom‐made cuvette consisting of a 6 cm^2^ gasket fixed to a piece of thin polyester stocking fabric (Figure S1, Perera‐Castro et al. 2020) and introduced in the Li‐6800 chamber, placed between the two gaskets of the chamber. The water concentration of the incoming air (reference water) was set to 0 mmol mol^−1^ air to enhance the dehydration of samples inside the chamber during the measurements and to avoid the risk of condensation in the air pathway. The flow rate within the chamber was maintained at 400 μmol s^−1^, and the CO_2_ concentration in the incoming air was set at 420 μmol mol^−1^ air. Irradiance was set at a saturation level of 800 μmol m^−2^ s^−1^ (tested previously with light‐response curves) for *A*_N_ measurement or switched off for at least 5–10 min for determination of *R*_D_. Gas exchange and chlorophyll fluorescence recordings were alternated with weighing of the sample each 5–10 min to obtain the fresh weight (FW) of the sample to complete the dehydration curve. An entire cycle of dehydration lasted for approximately 2 h with a similar dehydration rate between species that ranged from 97 to 250 mL H_2_O g^−1^ DW min^−1^ for the full range of the measured temperatures (15°C–35°C). Water content (WC) at any given time during dehydration was calculated as: WC = (FW‐DW)/DW, where DW is the dry weight obtained after keeping the samples at 70°C for 48 h. This procedure was repeated for different samples at several temperatures of the measuring chamber (15°C, 20°C, 25°C, 30°C and 35°C, 5 biological replicates each for *A*_N_ and *R*_D_) to obtain complete dehydration curves at each temperature. *A*_N_ and *R*_D_ were expressed per projected capitula area. In addition, the response of the maximum yield of PSII (*F*_v_/*F*_m_) to dehydration was also monitored for additional air‐dried samples at 25°C, following the same procedure described above under dark conditions.

Due to low gas exchange rates, especially *R*_D_, measurements of CO_2_ leakages (Flexas et al. 2007) were done regularly (each 15–30 min). Since relative humidity in the chamber varied depending on the hydration state of the sample (RH of 10%–80%), an additional correction was applied to avoid artefacts in the assimilation rate. A wet piece of paper was introduced in an empty moss cuvette, and CO_2_ of reference (CO_2_R) and sample (CO_2_S) were monitored during the dehydration curve using the same settings as for the measurements described above. The correction factor (*F*) used for correcting the CO_2_S was calculated as the ratio CO_2_R/CO_2_S, and its relationship with water vapour difference between sample and reference IRGAs (H_2_OS and H_2_OR, respectively) was obtained by linear regression (*R* ^2^ = 0.998, Figure S2):

(1) $$F=9.73\cdot 10^{-4}\cdot \left(H_{2}\mathrm{OS}–H_{2}\text{OR}\right)+1$$

The final formula for *A*_N_ was:

(2) $$A_{N}=\frac{\text{Flow}\left({\mathrm{CO}}_{2}R-{\mathrm{CO}}_{2}S\cdot F\right)}{\text{Area}}$$

Notice that the formula of *A*_N_ provided by LICOR already contains its own *F* for correcting the effect of air dilution by evaporated and/or transpired water. However, it was not enough for the high humidity levels of our chamber and the sensitivity requirements of our analysis.

### 2.3 In Situ Measurement of the Effect of Water Content and Irradiance on Moss Temperature

Temperature (*T*_moss_), WC, and irradiance (considering photosynthetic active radiation, PAR) were monitored in moss capitula during 3 days of diverse environmental conditions in the field. Moss canopy samples of 7 cm^2^ (*n* = 12) were extracted in their original structure using a 1 cm deep cylinder (Figure 1E). Moss and the cylinder were placed over a plastic plate near (< 5 m) their original population (Figure 1D). Both the cylinder and plate were weighted previously and accompanied the same sample during all measurements. *T*_moss_ was measured with an infra‐red thermometer (DT‐8666, CEM Instruments) and a K‐type thermocouple thermometer (TM‐902C UXCell) by pointing to or touching the surface of the central moss capitula. Temperatures obtained from the two instruments were very similar (Figure S3); therefore, *T*_moss_ was calculated as the average of both values. Immediately after *T*_moss_ determination, samples were weighed for calculating WC, as explained above, using a field portable balance (ML203E, Mettler Toledo International Inc.). PAR flux was measured with a QSPAR Quantum sensor (Hansatech Ltd.) before and after *T*_moss_ and WC determination. Both values of PAR (Figure S4) were averaged for subsequent analysis.

### 2.4 Modeling Net CO_2_ Assimilation

The response of *A*_N_ to radiation, water content, and temperature was fitted to a non‐linear model using the R statistical software (1.4.2.3 version) (R Core Team 2023). The semi‐empirical model was based on using the light response of photosynthesis and the exponential response of respiration as a base for net assimilation (Street et al. 2012; Calama et al. 2013; Günther et al. 2017; Silvan et al. 2017; Gong et al. 2020), with some modifications. Our model was based on a light response equation (E. L. Smith 1936) fitted to data:

(3) $$A_{N}=\frac{\left(A_{\mathrm{sat}}+R_{D}\right)\cdot \mathrm{PAR}\cdot \mathrm{AQE}}{\sqrt{{\left(\mathrm{AQE}\cdot \mathrm{PAR}\right)}^{2}+{\left(A_{\mathrm{sat}}+R_{D}\right)}^{2}}}-R_{D}$$

where *A*_sat_ is the light‐saturated *A*_N_ and AQE is the apparent quantum efficiency (initial slope of the light curve). Averaged AQE was considered as 0.0144 obtained from the light curves performed at 25°C, assumed to remain constant for 15°C–35°C, as reported in other moss species (Perera‐Castro et al. 2020, data available upon request). *A*_sat_ was expressed as a non‐symmetric polynomial function of *T*_moss_ and WC:

(4) $$A_{\mathrm{sat}}=a{T_{\text{moss}}}^{2}+bT_{\text{moss}}+c-\left(d{\mathrm{WC}}^{2}+\mathrm{eWC}\right)$$

where *a*, *b*, *c*, *d*, and *e* are fitted parameters. *R*_D_ was also expressed as a function of *T*_moss_ and WC following the empirical model of Heskel et al. (2016):

(5) $$R_{D}=e^{f+0.1012T_{\text{moss}}-0.0005{T_{\text{moss}}}^{2}}-\mathrm{gWC}+h+i\cdot \log\left(\mathrm{WC}\right)\cdot T_{\text{moss}}$$

where *f*, *g*, *h*, and *i* are fitted parameters.

The procedure to solve Equation (3) consisted of two independent steps. First, parameters *f*, *g*, *h*, and *i* were fitted from the temperature and water content response of the dataset obtained under dark conditions using Equation (5). Once these parameters were obtained, the measured *A*_N_, PAR, *T*_moss_, and WC were used to obtain model fittings for *a*, *b*, *c*, *d*, and *e* by combining Equations ((3), (4), (5)). Modelled *A*_N_ was thus calculated from PAR, *T*_moss_, WC, and the fitted parameters (*a–i*). This two‐step procedure avoided the undesirable effect of the different sizes of datasets (*R*_D_ vs. *A*_N_ under irradiated conditions) on the quality of the fitting.

A second model was developed to obtain *T*_moss_ from in situ WC, PAR, and air temperature (*T*_air_). *T*_air_ was obtained from meteorological data of the SMEAR II Hyytiälä forest meteorological station, available at the data portal of SMEAR (https://smear.avaa.csc.fi/download) (Figure S5). *T*_moss_ can be considered close to *T*_air_, plus the warming‐up resulted from PAR, which is mitigated by WC. Therefore:

(6) $$T_{\text{moss}}=T_{\mathrm{air}}+j{\mathrm{PAR}}^{2}+\text{kPAR}+l+m\cdot \log\left(\mathrm{WC}\right)$$

where *j*, *k, l* and *m* are fitted parameters.

Both *A*_N_ and *T*_moss_ model fits were carried out through ordinary nonlinear least squares regression techniques using the *nlme* package (Pinheiro et al., 2023). Additional R packages used for data analysis were *plyr* (Wickham 2011), *ggplot2* (Wickham 2016), and *agricolae* (de Mendiburu 2021).

### 2.5 Net Assimilation of *Sphagnum* Under Different Scenarios

Both *A*_N_ and *T*_moss_ models were combined to calculate the *A*_N_ of *S. squarrosum* and *S. angustifolium* for the natural environmental conditions and photoperiod of the studied period (from 3rd to 17th July 2023; SMEAR II Hyytiälä forest meteorological station) (Figure S5). Meteorological *T*_air_ and PAR were used for Equations (3 and 6) with some modifications to produce different scenarios: (1) variable water content (using WC values across the measured range, from 4.5 to 17 g H_2_O g^−1^ DW), reflecting variation in the water table from both flooding and drought situations, (2) variations of meteorological *T*_air_ of ±10°C under optimal WC, which corresponds to a 2‐fold change of the maximum predicted increase of mean *T*_air_ for Finland (Venäläinen et al. 2020), and (3) PAR flux variation under optimal WC. Since it is unknown how the reduction of total cloud cover (predicted to be −1.4% for the next 20 years, Mendoza et al. 2021) will affect integrated PAR flux, a reasonable ample range of variation of PAR was tested instead (variation factors of 0.6–1.6). Furthermore, *A*_N_ and *T*_moss_ models were used for calculating *A*_N_ of both studied species under two additional scenarios that may affect respiration losses: (4) variations in photoperiod (12/12, 18/6 or 20/4 h day/night photoperiods), and (5) changes in night temperatures (25°C/15°C, 25°C/20°C, or 25°C/25°C day/night temperatures). Scenarios 4 and 5 were modelled under optimum water content conditions and saturating light conditions (800 μmol m^−2^ s^−1^).

Since the meteorological station provided meteorological data (*T*_air_ and PAR) with a frequency of 30 min, daily net primary productivity of the studied period (*NPP*_day_; g CO_2_ m^−2^) was estimated from averaged meteorological conditions as:

(7) $${\mathrm{NPP}}_{\mathrm{day}}=\sum_{23:59}^{00:00}\frac{\text{modelled}\;A_{N}\cdot 1800\cdot {\mathrm{MW}}_{\mathrm{CO}2}}{10^{6}}$$

where *MW*_CO2_ is the molecular weight of CO_2_ (44.01 g mol^−1^). Notice that *NPP*_day_ refers to projected area of the capitula and not to the ground area. Since photosynthetic tissues are localized in the tips of the shoots, capitula are the main drivers of most CO_2_ assimilation of the plant and the primary productivity. Errors of the calculated *NPP*_day_ were obtained for the different scenarios by creating a bootstrapping dataset: the function *rnorm*() of the *stats* package (R Core Team 2023) was used for creating an artificial data frame of 1000 observations per fitted parameter from parameter *a* to *m*; Equations ((4), (5), (6)) with mean and standard deviation identical to the mean and standard deviation of each parameter. The combination of this bootstrapping data frame with the meteorological dataset allowed the calculation of 1000 values of *A*_N_ per any observation of *T*_air_ and PAR in the meteorological data (each 30 min). The average of those 1000 values of *A*_N_ allowed the calculation of the total standard deviation of the obtained *A*_N_ value per meteorological data. Then, as done for modelled *A*_N_, the obtained standard deviation was added for the summer period and transformed into g CO_2_ m^−2^. This procedure was repeated for all scenarios and the two species. Notice that for scenarios 4 and 5 the meteorological data was not used, and set values of PAR and temperature were used instead.

## 3 Results

### 3.1 *A*_N_ and *R*_D_ Response to WC and Temperature

**FIGURE 2:**
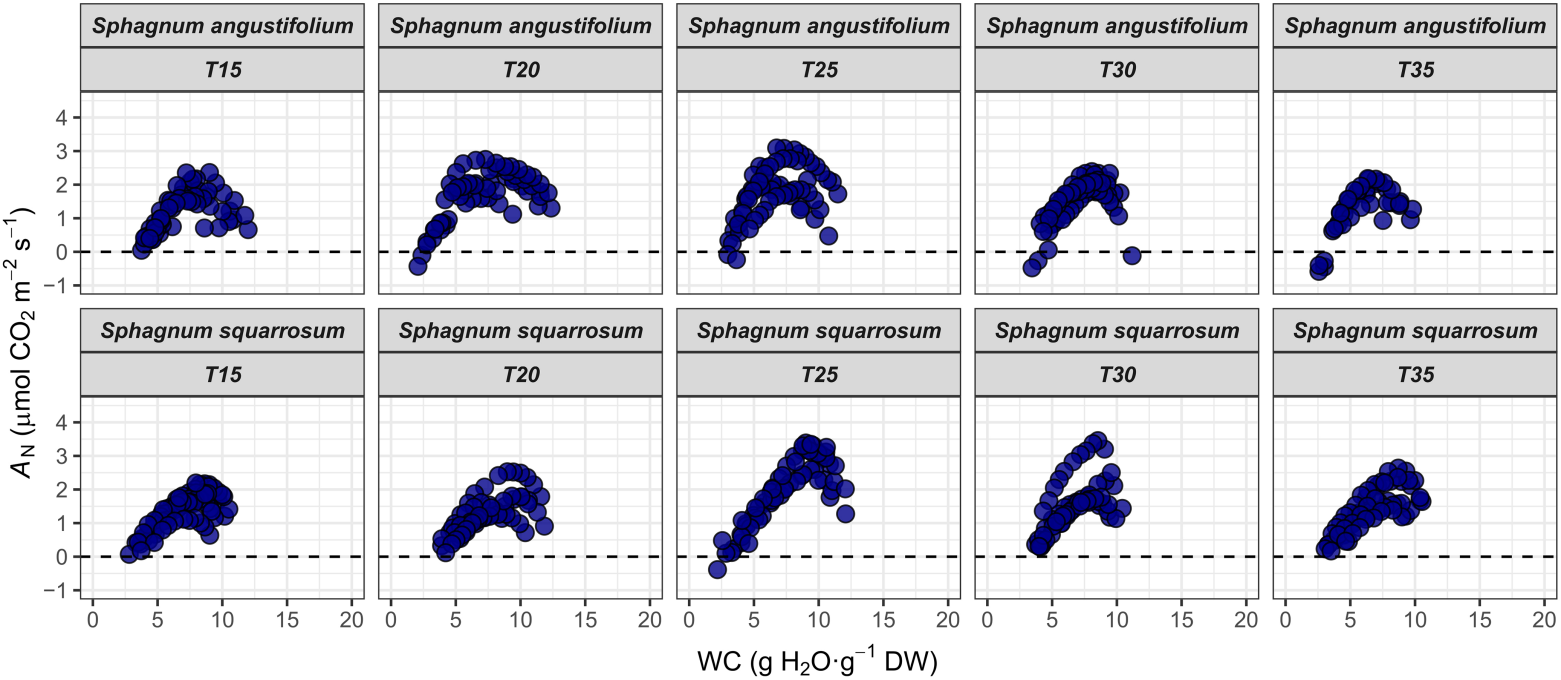
Net CO_2_ assimilation (*A*_N_) under saturated light conditions in response to water content (WC) of *S. angustifolium* (top panels) and *S. squarrosum* (bottom panels) during dehydration curves at 15°C, 20°C, 25°C, 30°C and 35°C (see Figure S6 for detailed response at 25°C).

**FIGURE 3:**
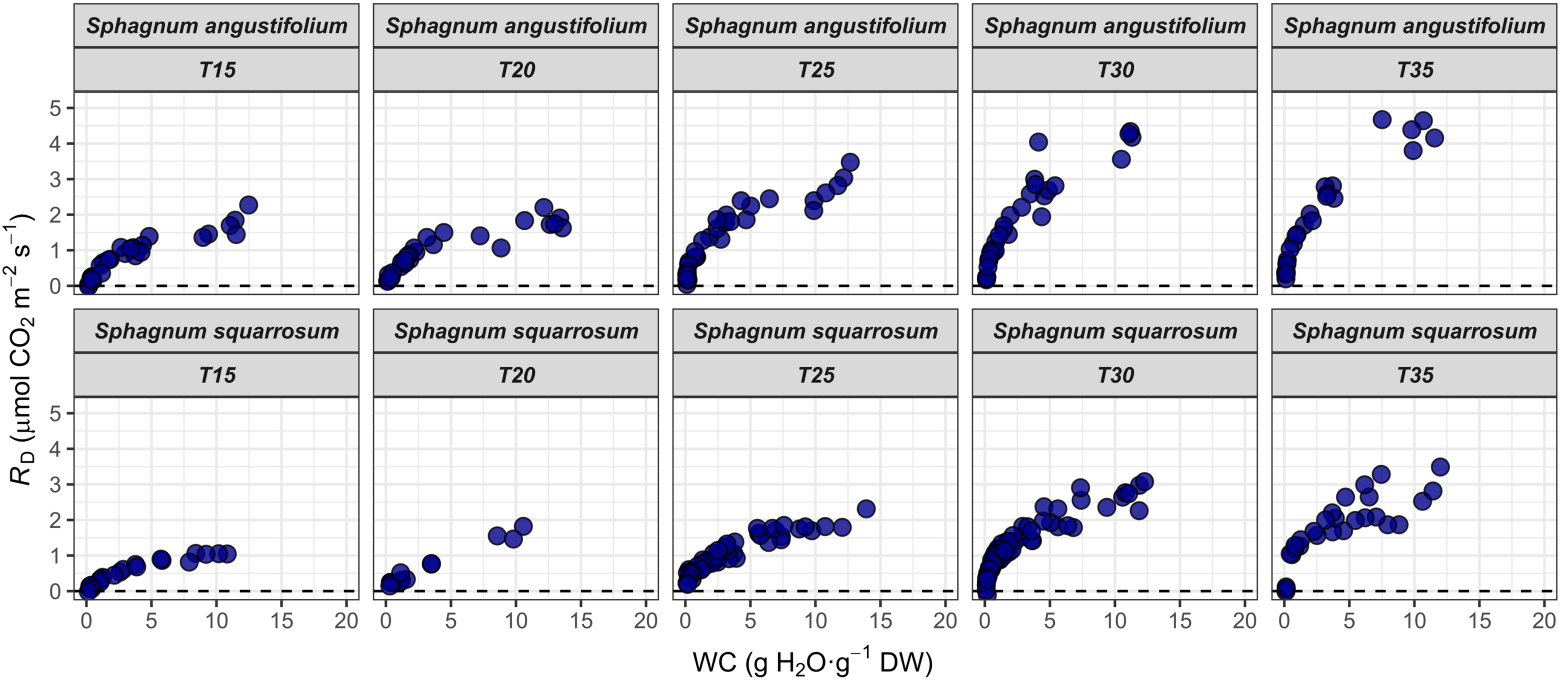
Dark respiration rates (*R*_D_) in response to water content (WC) of *S. angustifolium* (top panels) and *S. squarrosum* (bottom panels) during dehydration curves at 15°C, 20°C, 25°C, 30°C, and 35°C (see Figure S6 for detailed response at 25°C).

For all temperatures, *A*_N_ increased during the initial dehydration of the moss inside the chamber until a WC of 7.2 ± 0.3 and 8.6 ± 0.1 (mean ± SE) g H_2_O g^−1^ DW for *S. angustifolium* and *S. squarrosum* , respectively (Figures 2 and S6A,B for details at 25°C). On the contrary, *R*_D_ was not inhibited at high WC values (Figures 3 and S6C,D). Furthermore, both parameters differed in their x‐intercept. *A*_N_ was zero at WC, close to those at which *F*_v_/*F*_m_ remained at 50% of its non‐stressed value after a dehydration/rehydration cycle (Figures S6 and S7). At this point, *R*_D_ was reduced by 50% of the hydrated value and only reached zero at WC close to 0 g g^−1^ (Figure S6C,D).

**FIGURE 4:**
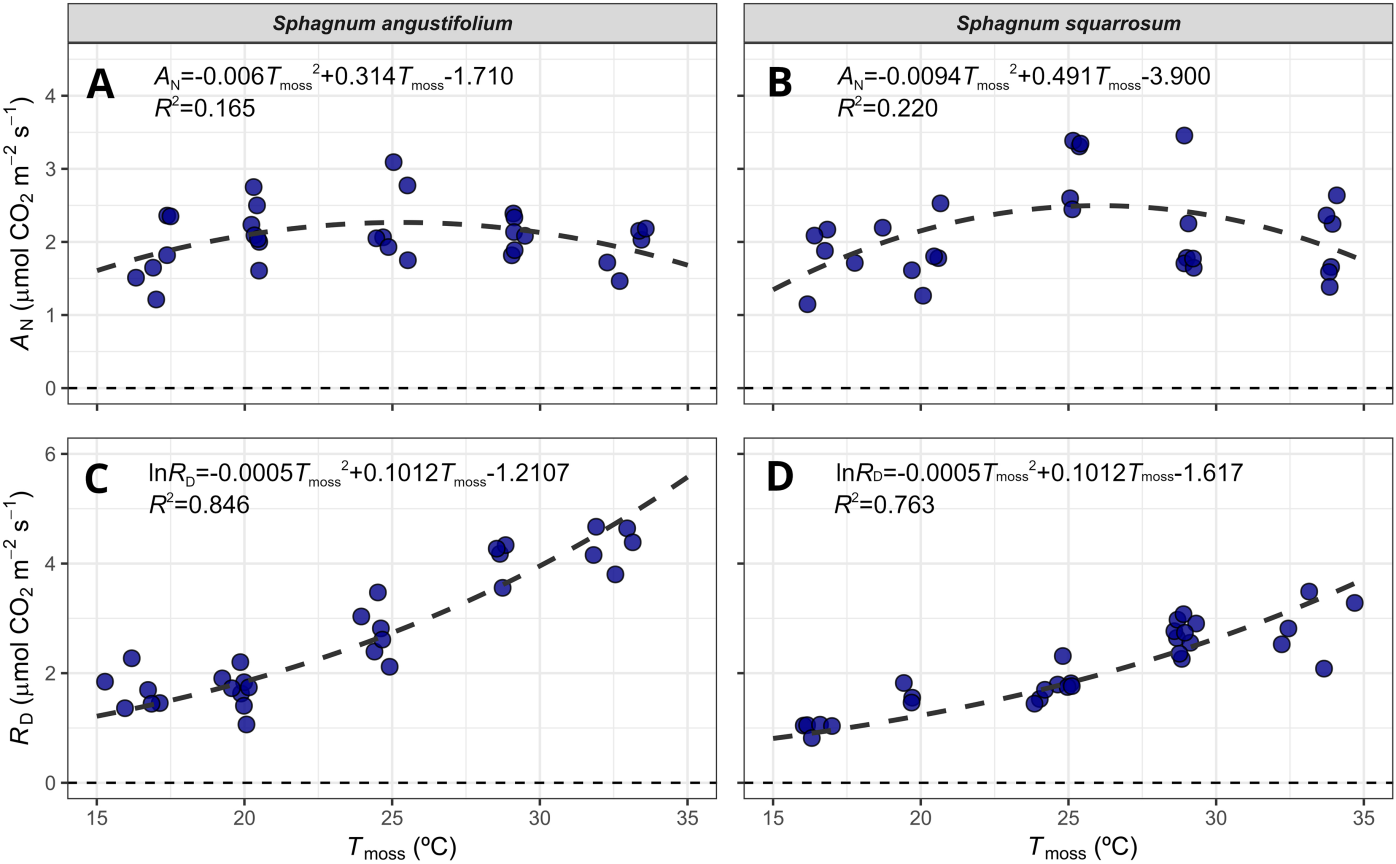
Maximum net CO_2_ assimilation (*A*_N_, A, B) under saturated light conditions and maximum dark respiration rates (*R*_D_, C, D) in response to temperature (*T*_moss_) of *S. angustifolium* (A, C) and *S. squarrosum* (B, D). Maximum *A*_N_ of each dehydration curve was obtained for a water content of 7.2 ± 0.3 and 8.6 ± 0.1 g H_2_O g^−1^ DW for *S. angustifolium* and *S. squarrosum* , respectively. Maximum *R*_D_ correspond to a water content of 11 ± 1.5 and 9.6 ± 1.9 g H_2_O g^−1^ DW for *S. angustifolium* and *S. squarrosum* , respectively. Dashed black lines represent a polynomial function for A and B, and the exponential function of Heskel et al. (2016) for C and D (*n* = 5).

Under optimum water content conditions (when *A*_N_ reached its maximum values in the dehydration curve), both species presented similar optimum temperatures for assimilation (Figure 4A,B). At 25°C, *Sphagnum squarrosum* presented a higher maximum *A*_N_ than *S. angustifolium* (2.8 ± 0.3 μmol m^−2^ s^−1^ vs. 1.8 ± 0.2 μmol m^−2^ s^−1^, *p* < 0.001, *t*‐test). *A*_N_ at 30°C and 3°C did not significantly differ from those at 25°C in both species. Regarding respiration, *R*_D_ increased exponentially with temperature (*R* ^2^ = 0.846 and 0.763 for *S. angustifolium* and *S. squarrosum* , respectively, with all fitted parameters significantly different to zero, Figure 4C,D). *R*_D_ above 27°C was significantly higher in *S. angustifolium* than in *S. squarrosum* (*p* < 0.001, *t*‐test, Figure 4C,D).

**FIGURE 5:**
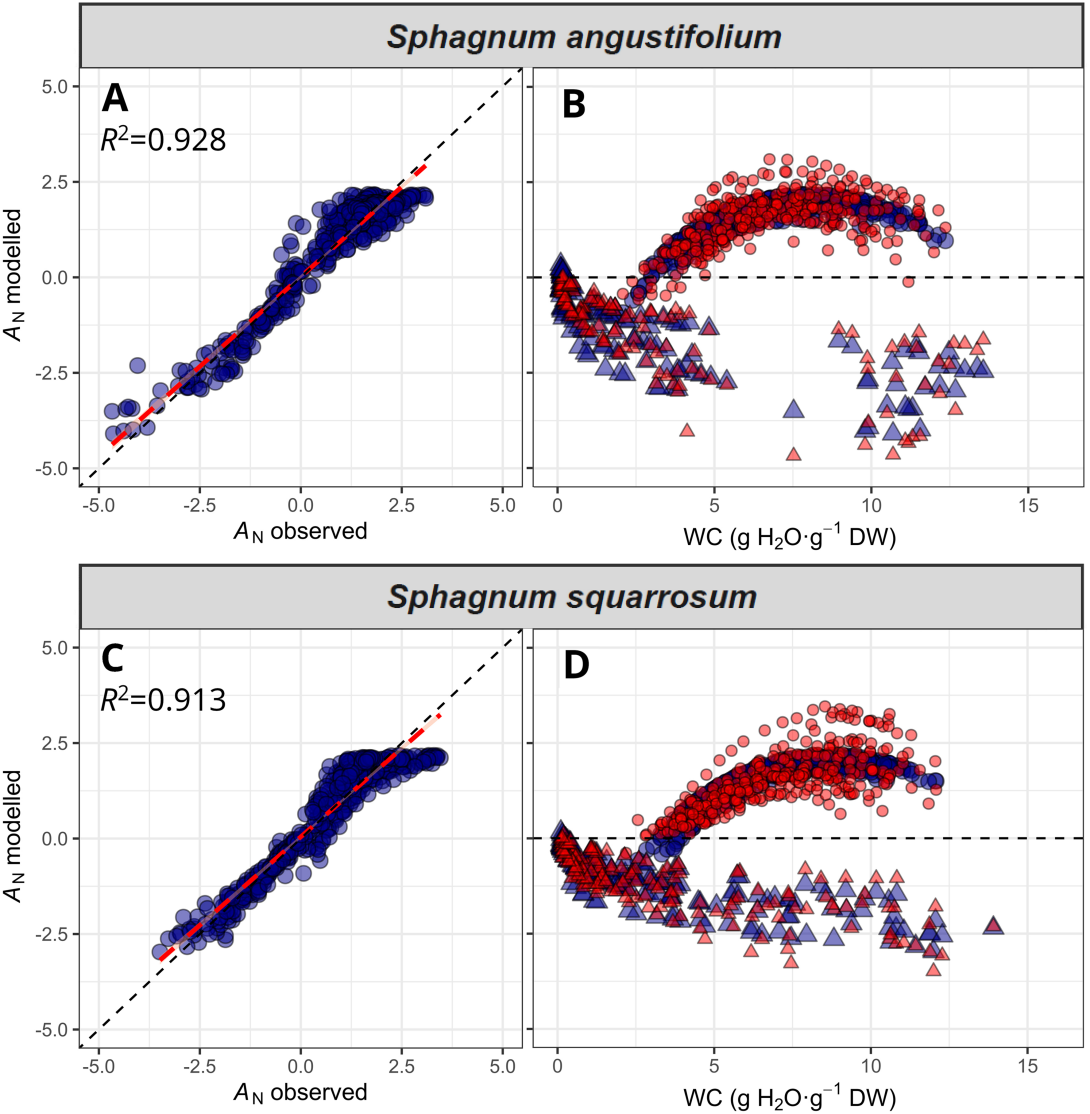
Relationship between modelled net CO_2_ assimilation rate (*A*_N_) and measured *A*_N_ of *S. angustifolium* (A, B) and *S. squarrosum* (C, D) considering dehydration curves for all temperatures and light conditions. Circles denote data under saturating light conditions (PAR of 800 μmol m^−2^ s^−1^) and triangles under dark conditions. Red dashed line indicates linear regression, which remains very close to the 1:1 relationship (black dashed line). Parameters fitted to both species are detailed in Table S1. In B and D panels, red datapoints indicate measured *A*_N_ and blue points indicate the estimated *A*_N_ under 800 μmol m^−2^ s^−1^ or dark conditions (equivalent to negative respiration: −*R*_D_) and blue points indicate the estimated *A*_N_ for the same conditions according to the model (Equations (3), (4), (5)). The final total number of recordings used in the model were 431 and 498 for *S. angustifolium* and *S. squarrosum* , respectively.

When all data (WC curves at 5 temperatures) were fitted to the described *A*_N_ model, good fits were obtained for *S. angustifolium* (*R* ^2^ = 0.928, Figure 5A,B) and *S. squarrosum* (*R* ^2^ = 0.913, Figure 5C,D). The values of the parameters fitted for the *A*_N_ model of both species are shown in Table S1.

### 3.2 Drivers of Moss Canopy Temperature

**FIGURE 6:**
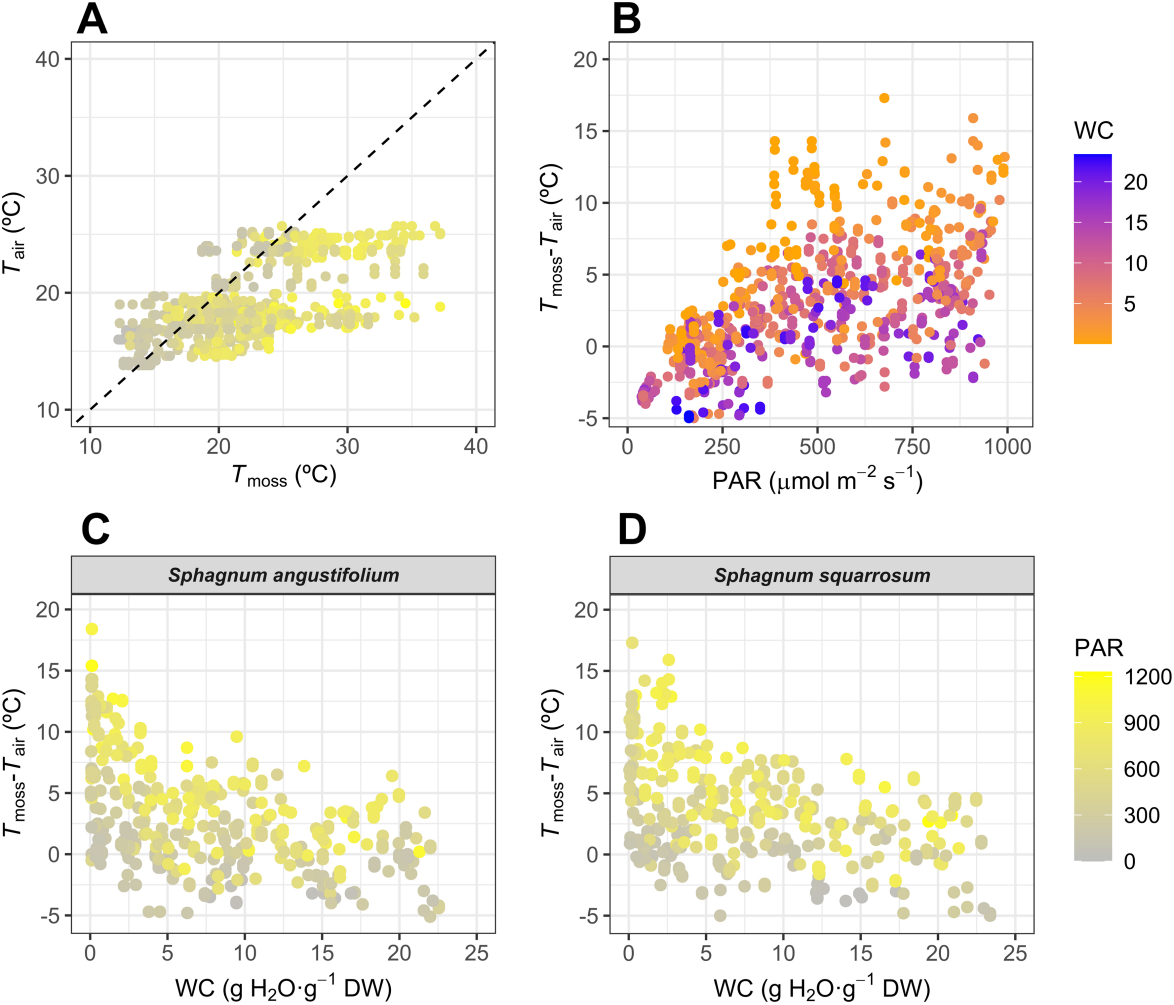
(A) Relationship between measured moss canopy temperature (*T*_moss_) and air temperature of the meteorological station SMEAR II Hyytiälä forest, Finland (*T*_air_) in relation to photosynthetic active radiation (PAR). (B) Warming of moss canopy (*T*_moss_–*T*_air_) in relation to PAR and water content (WC). Data for both species are presented together in (A, B). Variation of moss canopy temperature in relation to moss water content (WC) and photosynthetic active radiation (PAR) of *S. angustifolium* (C) and *S. squarrosum* (D).

**FIGURE 7:**
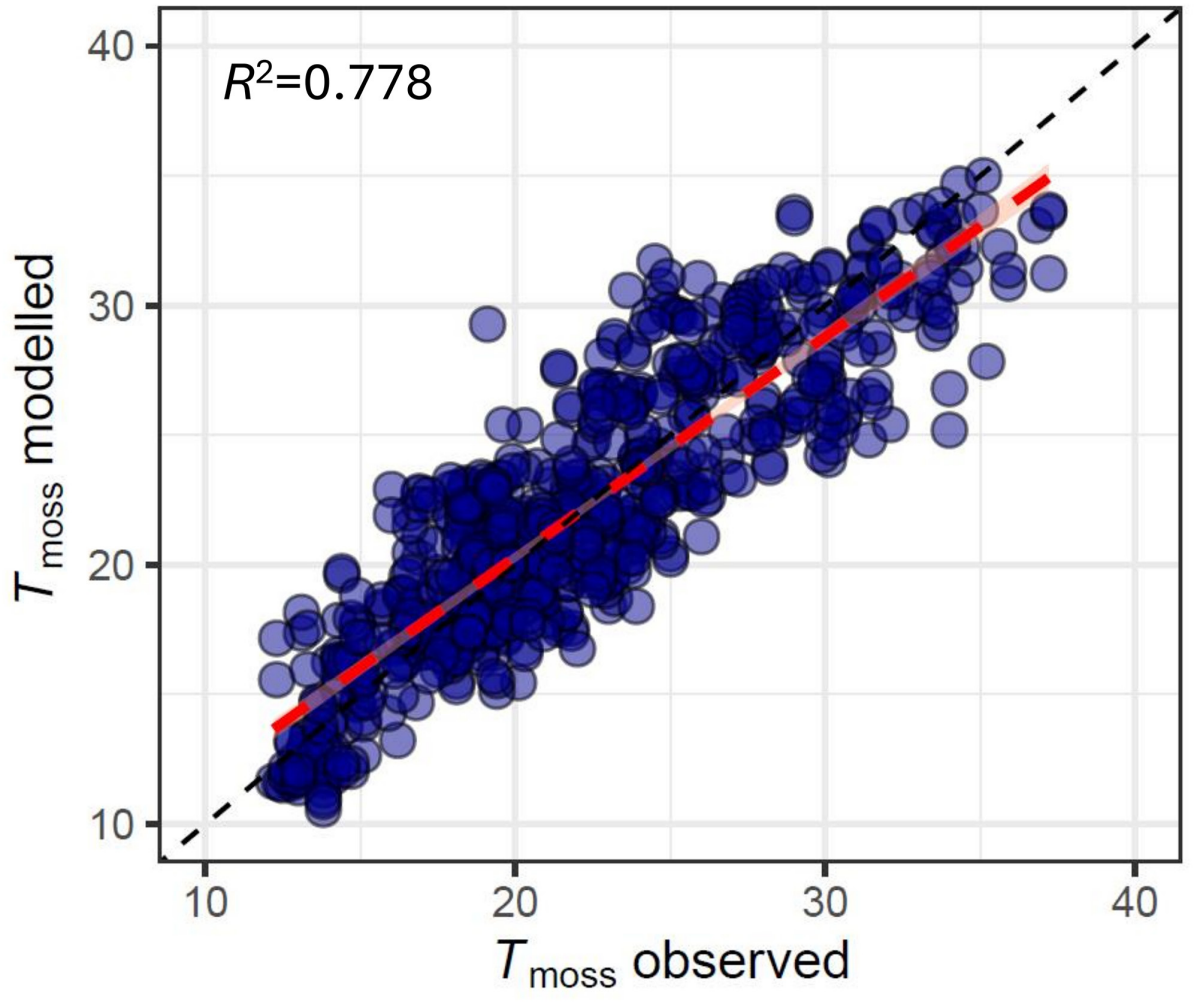
Relationship between measured and modelled moss canopy temperature (*T*_moss_). Data for *S. angustifolium* and *S. squarrosum* were considered together for this analysis. Red dashed line indicates linear regression, which corresponds to the 1:1 relationship (black dashed line). The fitted model is: *T*_moss_ = *T*_air_ + 0.017·PAR –8.2·10^−6^·PAR^2^ –1.624·*log*(WC)–0.734, where WC is the water content of the sample, PAR is the photosynthetic active radiation measured in the field, and *T*_air_ the air temperature registered by the SMEAR II Hyytiälä forest meteorological station. SD and significance of fitted parameters are detailed in Table S2.

A wide range of *T*_moss_ values was measured in the field (*T*_moss_ between 12°C–37°C), greater than the *T*_air_ range registered by the meteorological station for the same measuring time (13°C–26°C) (Figure 6A,B). For all WC values, the highest differences between *T*_moss_ and *T*_air_ (15°C maximum) were registered at the highest PAR values and the lowest WC in both species (Figure 6B). The effect of WC in buffering the warming of the moss canopy was only relevant at WC > 5 g H_2_O g^−1^ DW (Figure 6C,D), when the corresponding *A*_N_ of both species had already decreased more than 50% of optimum values, and *F*_v_/*F*_m_ started decreasing in an irreversible way (Figures S6 and S7). When data of both species were combined and fitted to the *T*_moss_ model (Table S2), a good fit was obtained when comparing observed and modelled temperatures (*R* ^2^ = 0.778, Figure 7), although 22.2% of total variance remained unexplained. In contrast, for modelled *A*_N_, only 7.2%–8.7% of variance remained unexplained (Figure 5A,C).

### 3.3 Scenarios of Carbon Balance

**FIGURE 8:**
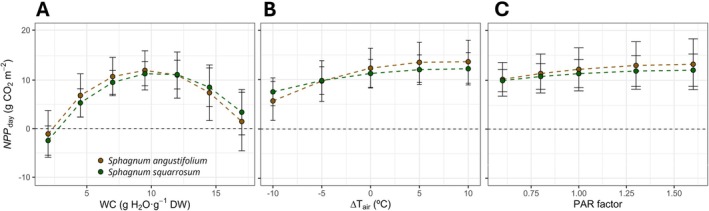
Daily net primary productivity of summer (3rd–17th July, 2023) (*NPP*_day_) estimated from the combination of the *A*_N_ and *T*_moss_ models and different scenarios: (A) complete range of measured WC (scenario 1), (B) variations of each recorded meteorological *T*_air_ from −10°C to +10°C under optimal WC (scenario 2), and (C) PAR variation factors projected for the next decades, including those modelled by Mendoza et al. (2021) in the 0.6–1.6 range, under optimal WC (scenario 3). Datapoints are mean ± SD. Standard deviation was calculated by generating a bootstrapping dataset of mean ± SD of each fitting parameter.

Combining the *A*_N_ and *T*_moss_ models (Equations (3), (4), (5), (6)), *A*_N_ was estimated from meteorological data of SMEAR II Hyytiälä forest station, and *NPP*_day_ was approximated from the integration of modelled *A*_N_ during the summer period (3rd to 17th July). *T*_air_ values registered for the studied period at SMEAR II Hyytiälä forest station oscillated between averaged daily minimum *T*_air_ of 12.4°C ± 1.1°C during the night at 03:00 h to averaged maximum *T*_air_ of 19.4°C ± 3.5°C during midday (14:00 h) (Figure S5). The average daily maximum PAR at midday was 750 μmol m^−2^ s^−1^ (Figure S5) during a day length of 20.5 h. Under these conditions, maximum *NPP*_day_ at the optimum values of WC were estimated as 12.0 ± 4.0 and 11.3 ± 2.4 g CO_2_ m^−2^ for *S. angustifolium* and *S. squarrosum* , respectively (Figure 8A). Under optimum WC conditions, an increase of 5°C above the *T*_air_ (and therefore *T*_moss_, depending on PAR) resulted in an enhancement of mean *NPP*_day_ of only 1.1 and 0.7 g CO_2_ m^−2^ for *S. angustifolium* and *S. squarrosum*, respectively (Figure 8B), without a decrease of *NPP*_day_ with +10°C of the reported temperatures. Something similar was observed for the estimation of *NPP*_day_ under different reduction factors of PAR (and, therefore, subsequent effects on *T*_moss_). A PAR variation factor of 1.3 (PAR increase by 30%) only resulted in increases of average *NPP*_day_ by 0.7 and 0.5 g CO_2_ m^−2^ for *S. angustifolium* and *S. squarrosum* , respectively, under optimum WC (Figure 8C).

**FIGURE 9:**
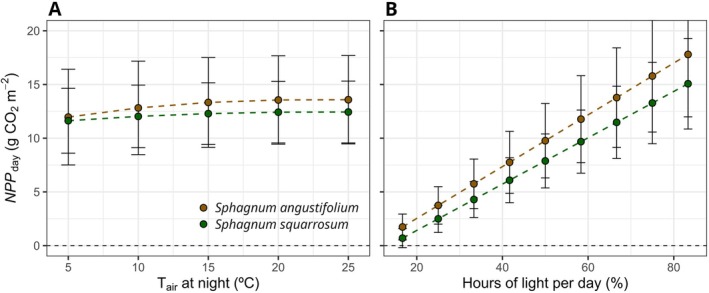
Estimated average daily net primary productivity (*NPP*_day_) under optimum conditions of water content for: (A) different air temperatures (*T*_air_) during night, considering a photoperiod of 20/4 h (day/night), optimum *T*_air_ (25°C) during day and saturating light conditions (800 μmol m^−2^ s^−1^) (scenario 4); and (B) different photoperiods represented as a percentage of hours per day considering an oscillation of *T*_air_ of 25°C/15°C (the natural one for the study site during summer) and saturating light conditions during day (scenario 5). Datapoints are mean ± SD. Standard deviation was calculated by generating a bootstrapping dataset of mean ± SD of each fitting parameter.

Our model also revealed a slight variation of averaged *NPP*_day_ with decreases of night temperature from 25°C to 5°C at optimum WC (1.6 and 0.8 g CO_2_ m^−2^ for *S. angustifolium* and *S squarrosum*) (Figure 9A). On the contrary, this averaged *NPP*_day_ increased by 8 and 7.2 g CO_2_ m^−2^ in *S. angustifolium* and *S. squarrosum* , respectively, if a short‐night photoperiod of 20/4 (day/night = 83% of hours of light per day) is modelled instead of 12/12 h (50% of hours of light) (Figure 9B).

## 4 Discussion

### 4.1 Source of Errors and Reliability of the Estimated *NPP*

The presented models calculated net CO_2_ assimilation in two *Sphagnum* species in response to radiation, temperature and water content, also considering the interaction between these variables in the determination of moss canopy temperature. Using a bootstrapping dataset of each fitting parameter, the total SD was calculated for the estimated *NPP*_day_ (3rd–17th July) for the two studied *Sphagnum* species. Most of the deviation originated in the estimation of *T*_moss_ from *T*_air_, radiation and WC, which was not as accurate as the estimation of *A*_N_ (Figures 4 and 6). Other factors may also affect *T*_moss_ and its interactions with radiation and WC, such as the wind speed (Bilgili 2010; Perera‐Castro et al. 2020), canopy structure (Rice et al. 2018), light angle (Fuchs 1990, for leaf temperature) or water temperature surrounding mosses, some of which are not easily accessible from meteorological stations. The measurement of water content in capitula in the field or under lab conditions necessarily requires the use of detached samples excised from the underlying tissue. Although this hinders direct comparison of intact *Sphagnum* shoots, continuously ‘watered’ by the lower layers of moss canopy, our main intent was to induce sample dehydration to measure temperature, *A*_N_ and *R*_D_ under the whole range of water content. Since the density of shoots and structure of the canopy was maintained for the detached 1 cm deep samples, we consider that the response of *T*_moss_ is comparable to that of the intact moss canopy, which is expected to experience fewer WC variations (Silvola and Aaltonen 1984; Gaberščik and Martinčič 1987; Kellner and Halldin 2002; Fukuta et al. 2012).

In the modelling of net assimilation, 7.2% and 8.7% of variance remained unexplained when compared to measured *A*_N_ in *S. angustifolium* and *S. squarrosum*, respectively, which may reflect the variability of plant material in the field, similar to other studies that modelled gas exchange fluxes in *Sphagnum* (Beyer and Höper 2015). The factors that explain this intraspecific variation in a single location remain unknown, possibly related to genetic variation, phenology, or stressful conditions prior to our measurements. The heterogeneity of ground level observed in the field is an indicator of different distances to the water table, which also can affect both biomass density and moss growth (Serk et al. 2021). Serk et al. (2021) also reported a growth increase due to short‐term exposure to high CO_2_ concentration (8–10 weeks under 280 vs. 400 ppm CO_2_), although the increase of CO_2_ exchange at enhanced CO_2_ is expected to be dissipated after long‐term exposure (> 17 weeks, Jauhiainen and Silvola 1999). The effect of long‐term exposure to other factors, such as stressful temperatures or unfavourable WC, may explain seasonal decreases in the photosynthetic capacity of *Sphagnum* (Gaberščik and Martinčič 1987; Fukuta et al. 2012). Heat waves can also compromise the photosynthetic capacity and yield of *Sphagnum* (Gerdol and Vicentini 2011), probably followed by a delayed recovery. Fukuta et al. (2012) also reported that the relative response of *A*_N_ to water content and temperature did not change seasonally, although possible acclimation in other *Sphagnum* species could still play a role throughout the year. Our models were not conceived to estimate annual NPP. However, their good fit to the measured *A*_N_ data makes them suitable for estimating changes in the maximum optimum *NPP*_day_ of the growing season and estimating the average balances between CO_2_ uptake and respiration in the different tested scenarios.

### 4.2 Water Content as a Double‐Edged Sword

In the field, *Sphagnum* tissues are often far from their optimal water content for assimilation, being either overhydrated or desiccated–a dynamic state typical of poikilohydric plants (Proctor et al. 2007). Dehydration below optimum WC can lead to irreversible damage to the photosynthetic apparatus, reflected by the decrease in *F*_v_/*F*_m_ (Figure S7), although some authors have reported recovery after extreme water loss (i.e., desiccation tolerance) in *Sphagnum* (Schipperges and Rydin 1998; Hájek and Beckett 2008), probably associated with dehydration rate and ABA signalling (Marschall and Borbély 2011; Hájek and Vicherová 2014). *Sphagnum* tissues and canopy structure allow for a desiccation avoidance strategy, enhancing the retention of a considerable reservoir of water (the highest among bryophytes; Wang and Bader 2018) inside porous, hyaline, non‐photosynthetic cells and capillary spaces formed by overlapping pendent branches that hang down against the stem (Van Breemen 1995; McCarter and Price 2014). Since CO_2_ diffuses about 10,000 times more slowly in water than in air (Griffiths et al. 2004), this high capacity of *Sphagnum* for storage water constrains CO_2_ internal conductance and photosynthetic capacity under WC higher than optimum (Dilks and Proctor 1979; Proctor 2000; Rice et al. 2011; Perera‐Castro, Waterman, et al. 2022). Furthermore, the *Sphagnum* structure also allows water movement by capillary action from the water table to *Sphagnum* capitula at higher levels (Van Breemen 1995), something that varies between species, as well as their preferred height above the water table (Laing et al. 2014; Bengtsson et al. 2016; Piatkowski and Shaw 2019).

Water levels in peatlands can vary seasonally and annually (Kurnianto et al. 2015; Swindles et al. 2015; Silvan et al. 2017; Karki et al. 2019; Sim et al. 2021). Several studies have revealed a relationship between water level and NPP (Gunnarsson 2005; Walker et al. 2017; but also Street et al. 2012). However, due to the complex regulation of CO_2_ and CH_4_ release by heterotrophic respiration, the response of net ecosystem productivity to water level seems elusive (Updegraff et al. 2001; Flanagan and Syed 2011), with possible enhanced methane emissions if the water level reaches the ground level (Beyer and Höper 2015). Cases of success in farming *Sphagnum* are based on tight control of irrigation for enhancing moss growth (examples in Cris et al. 2011, 2014; Günther et al. 2017; Gaudig et al. 2018; Grobe et al. 2021). Our results show that NPP could be enhanced considerably if the water table is controlled to allow optimum water content for gas exchange and follows the increase in height of moss shoots to avoid chlorosis and dehydration for the highest shoots (Hoshi 2017). That may avoid overhydration and problems with CO_2_ diffusion and methane production (Beyer and Höper 2015). Monitoring evapotranspiration (Moore et al. 2013) and the water table–WC and NPP–height relationship of the different *Sphagnum* species will be of great interest.

### 4.3 There Are Different Temperature Thresholds for Sink Capacity of Peatlands and *Sphagnum*

Mean annual temperature is frequently considered an important factor determining productivity on a global scale in *Sphagnum*‐dominated wetlands (Gunnarsson 2005). Most studies that estimate the effects of global warming on the carbon balance of peatlands agree that their sink capacity could be substantially reduced by 2050 under the high‐warming scenario (RCP8.5) due to increases in mineralization rates (Chaudhary et al. 2020), becoming even a source of CO_2_ (Qiu et al. 2020; Hanson et al. 2020). In addition, a risk of shifting wetlands from a wet system dominated by cryptogams to a drier system with increased cover of vascular plants has also been predicted (Bao, Jia, and Xu 2022). However, Charman et al. (2013) predicted an increase in the carbon sequestration rate of peatlands in a climate warming scenario, but only if water availability is maintained. In their approach, PAR dose (daily radiation level on days when the temperature is above 0°C) had a positive effect on carbon accumulation. Lunt et al. (2019) reported the same past and contemporary high rates of carbon accumulation for a peatland in South West England, suggesting that recent climate changes appeared to have had no impact on their role as carbon sinks. In agreement with these studies, our estimations of NPP_day_ from the modeled *A*_N_ using meteorological data for the summer (and, therefore, not considering heterotrophic respiration), reflect a potential suboptimal carbon sink capacity that can be slightly enhanced (by 1.1 and 0.7 g CO_2_ m^−2^ for *S. angustifolium* and *S. squarrosum* , respectively) if each registered *T*_air_ value increases by 5°C (Figure 8B). Notice that this change in *T*_air_ has an amplified effect in *T*_moss_, which would increase by 6.1°C and 5.7°C in *S. angustifolium* (WC = 9.5) and *S. squarrosum* (WC = 10.75) during the midday in sunny days, according to the *T*_moss_ model. *S. angustifolium* and *S. squarrosum* presented optimum temperatures for the highest *A*_N_, around 25°C, with a wide plateau. Meanwhile, the average diurnal *T*_air_ for the 2023 summer period at SMEAR II Hyytiälä forest station was 19.4°C ± 3.5°C, and 12.4°C ± 1.1°C during the night. Our data reflect that, under this context, an increase in temperature can enhance daily carbon fixation in photosynthesis more than carbon release in night respiration, at least for the first averaged 5° of increase.

Different predictions between studies of peatland sink capacity in warming scenarios may be related to different sink‐source thresholds for the components of net ecosystem productivity (NPP and heterotrophic respiration and mineralization). Hanson et al. (2020) estimated that an increase in mean annual temperature of 4°C will result in a negative net carbon balance due to an enhanced heterotrophic release of CO_2_ and CH_4_. However, our estimated *Sphagnum NPP*_day_ will not become negative even after a *T*_air_ increase of 10°C (Figure 8B). This suggests that the change of peatlands from sink to source of CO_2_ under warming scenarios may precede the *Sphagnum* optimum temperatures for *NPP*. Thus, optimum environmental conditions for maximizing *Sphagnum* CO_2_ fixation may at the same time be detrimental due to a concomitant increase in heterotrophic release of CO_2_ and CH_4_, transforming the ecosystem into a source of carbon even when its principal primary producers perform at their highest capacity.

### 4.4 Photoperiod Could Be the Main Limiting Factor for NPP

Although low night temperatures can reduce carbon losses due to respiration (with possibly some non‐temperature dependent changes in respiration rate, Bruhn et al. 2022), the model shows that an increased carbon gain results from shorter night periods (Figure 9). The relevance of respiration in determining NPP could explain the lack of correlation between CO_2_ assimilation and growth reported for *Sphagnum* (Gaberščik and Martinčič 1987). This result, together with the described suboptimal growth of *Sphagnum* in the study site (enhanced by warming), suggests photoperiod as a relevant driver of *Sphagnum* productivity and the global distribution of *Sphagnum*‐dominated mires, although intercorrelated with low temperatures (Campbell et al. 2021) and latitude (Tanneberger et al. 2017). If true, this new idea will involve that the affinity of mosses for high latitudes would be more related to shorter nights during the growing season –and, therefore, enhancing carbon balance–than with an intrinsic cold‐adapted physiology, as their relatively high optimum temperature for maximum *A*_N_ evidence (Figure 4, Perera‐Castro, González‐Rodríguez, and Fernández‐Marín 2022). *Sphagnum* is a widespread genus with an ample world distribution (Daniels and Eddy 1990). However, *Sphagnum*‐dominated bogs are more frequent in the northern hemisphere, while *Sphagnum*‐dominated peatlands of the tropics are less extensive and have a lower pool of C (Page and Baird 2016). Since most predictions of the spatial–temporal variation of *Sphagnum* (Ma et al. 2022) and mosses in general (He et al. 2016) are based on their cold affinity, considering the “photoperiod” hypothesis would be essential for understanding the response of the peatland ecosystem to global warming and also improve harvesting protocols.

Continuous light is sometimes used for in vitro cultivation of *Physcomitrella patens* (Calcutt et al. 2024). However, at least in some vascular plants, long‐term continuous light can be damaging (Velez‐Ramirez et al. 2011). Furthermore, the warming effect of sun radiation in enhancing *NPP*_day_ for scenarios with higher PAR, even in light‐saturated *Sphagnum* (Figure 8C), contradicts the observed enhancement of photosynthesis and yield when *Sphagnum* grows under shaded conditions (Murray et al. 1993; Hájek et al. 2009). Long‐term high light exposure, although positive in its warming effect and carbon balance, could have a negative effect on *Sphagnum* photosynthesis if photoinhibition is triggered. Future studies about the importance of the night period in the daily cycle of *Sphagnum* will shed light on the best settings for optimizing the culturing of this important moss and provide useful tools to monitor the future of peatlands.

## 5 Conclusions

The present study establishes the photosynthesis and respiration responses of *S. angustifolium* and *S. squarrosum* to tissue water content and temperature in a short period of the growth season (3rd–17th July). Net assimilation was quite sensitive to water content in both species. Notably, *A*_N_ presented an optimum around 25°C, while *R*_D_ increased significantly over the same temperature range. When *A*_N_ is integrated to calculate net primary productivity, our assimilation model reflects how the carbon fixation of *Sphagnum* can potentially be affected under certain scenarios. The simulation accounting for higher temperatures enhanced the estimated *NPP* of the two studied species if water content remained near optimum. In addition to water availability, the reduction of carbon losses by respiration during the night was also important for obtaining a positive daily carbon balance in our model. Thus, even when considering optimum temperatures of 25°C, when maximum assimilation rates were recorded, big variations in the *NPP* of both *Sphagnum* species were estimated in response to variations of the photoperiod due to reduced or enhanced carbon losses by respiration.

## Author Contributions

A.V.P.‐C. and M.N. designed the experiment, applied for the funding, and conducted the experiments; A.V.P.‐C. performed the analysis and wrote the first draft; both authors contributed to the final version of the manuscript.

## Supporting information


**Figure S1.** Custom‐made moss cuvette for moss placement during gas exchange measurement.
**Figure S2.** Linear regression between the CO_2_ sample factor and the water vapour difference between sample and reference IRGAs.
**Figure S3.** Relationship between moss canopy temperature (*T*_moss_) of the two used sensors: infra‐red sensor and thermocouple.
**Figure S4.** Relationship between PAR measured at the beginning of the cycle of moss temperature measurements and at the end of each cycle.
**Figure S5.** Air temperature and photosynthetic active radiation from 15 May to 15 September 2023 of SMEAR II Hyytiälä forest meteorological station.
**Figure S6.** Net CO_2_ assimilation under light conditions and dark respiration in response to water content during a dehydration curve at 25°C.
**Figure S7.** Response of maximum yield of PSII to water content of samples during air‐dehydration.
**Table S1.** Fitted parameters of the *A*_N_ Model (Equations (3), (4), (5)) for estimating net CO_2_ assimilation rates of *S. angustifolium* and *S. squarrosum* .
**Table S2.** Fitted parameters of the Model 2 (Equation 6) for estimating moss canopy temperature from PAR, water content and air temperature.

## Data Availability

The datasets used and/or analyzed during the current study are available at Zenodo (https://doi.org/10.5281/zenodo.15463352; Perera‐Castro and Nadal 2025) and from the corresponding author, Alicia V. Perera‐Castro, on reasonable request.

## References

[ppl70325-bib-0001] Asemaninejad, A. , R. G. Thorn , B. A. Branfireun , and Z. Lindo . 2018. “Climate Change Favours Specific Fungal Communities in Boreal Peatlands.” Soil Biology and Biochemistry 120: 28–36.

[ppl70325-bib-0002] Bao, T. , G. Jia , and X. Xu . 2022. “Warming Enhances Dominance of Vascular Plants Over Cryptogams Across Northern Wetlands.” Global Change Biology 28: 4097–4109. 35364612 10.1111/gcb.16182

[ppl70325-bib-0003] Bates, J. W. 1994. “Responses of the Mosses *Brachythecium Rutabulum* and *Pseudoscleropodium Purum* to a Mineral Nutrient Pulse.” Functional Ecology 8: 686–693.

[ppl70325-bib-0004] Bengtsson, F. , G. Granath , and H. Rydin . 2016. “Photosynthesis, Growth, and Decay Traits in Sphagnum–a Multispecies Comparison.” Ecology and Evolution 6: 3325–3341. 27103989 10.1002/ece3.2119 PMC4833502

[ppl70325-bib-0005] Bengtsson, F. , H. Rydin , J. L. Baltzer , et al. 2021. “Environmental Drivers of *Sphagnum* Growth in Peatlands Across the Holarctic Region.” Journal of Ecology 109: 417–431.

[ppl70325-bib-0006] Bergeron, O. , H. A. Margolis , and C. Coursolle . 2009. “Forest Floor Carbon Exchange of a Boreal Black Spruce Forest in Eastern North America.” Biogeosciences 6: 1849–1864.

[ppl70325-bib-0007] Beyer, C. , and H. Höper . 2015. “Greenhouse Gas Exchange of Rewetted Bog Peat Extraction Sites and a *Sphagnum* Cultivation Site in Northwest Germany.” Biogeosciences 12: 2101–2117.

[ppl70325-bib-0008] Bilgili, M. 2010. “Prediction of Soil Temperature Using Regression and Artificial Neural Network Models.” Meteorology and Atmospheric Physics 110: 59–70.

[ppl70325-bib-0009] Block, W. , R. I. Lewis Smith , and A. D. Kennedy . 2009. “Strategies of Survival and Resource Exploitation in the Antarctic Fellfield Ecosystem.” Biological Reviews 84: 449–484. 19659886 10.1111/j.1469-185X.2009.00084.x

[ppl70325-bib-0010] Bruhn, D. , F. Newman , M. Hancock , et al. 2022. “Nocturnal Plant Respiration Is Under Strong Non‐Temperature Control.” Nature Communications 13: 5650. 10.1038/s41467-022-33370-1 PMC9512894 36163192

[ppl70325-bib-0011] Calama, R. , J. Puértolas , G. Madrigal , and M. Pardos . 2013. “Modeling the Environmental Response of Leaf Net Photosynthesis in *Pinus pinea* L. Natural Regeneration.” Ecological Modelling 251: 9–21.

[ppl70325-bib-0012] Calcutt, R. , Y. Aghli , T. Arinzeh , and R. Dixit . 2024. “A Fibrous Scaffold for In Vitro Culture and Experimental Studies of *Physcomitrium Patens* .” Plant Direct 8: e570.

[ppl70325-bib-0013] Campbell, C. , G. Granath , and H. Rydin . 2021. “Climatic Drivers of *Sphagnum* Species Distributions.” Frontiers of Biogeography 13: e511.

[ppl70325-bib-0014] Charman, D. J. , D. W. Beilman , M. Blaauw , et al. 2013. “Climate‐Related Changes in Peatland Carbon Accumulation During the Last Millennium.” Biogeosciences 10: 929–944.

[ppl70325-bib-0015] Chaudhary, N. , S. Westermann , S. Lamba , et al. 2020. “Modelling Past and Future Peatland Carbon Dynamics Across the Pan‐Arctic.” Global Change Biology 26: 4119–4133. 32239563 10.1111/gcb.15099

[ppl70325-bib-0016] Convey, P. , S. J. Coulson , M. R. Worland , and A. Sjöblom . 2018. “The Importance of Understanding Annual and Shorter‐Term Temperature Patterns and Variation in the Surface Levels of Polar Soils for Terrestrial Biota.” Polar Biology 41: 1587–1605.

[ppl70325-bib-0017] Cove, D. J. , P. F. Perroud , A. J. Charron , S. F. McDaniel , A. Khandelwal , and R. S. Quatrano . 2009. “Culturing the Moss *Physcomitrella Patens* .” Cold Spring Harbor Protocols 4: 1–7. 10.1101/pdb.prot5136. 20147066

[ppl70325-bib-0018] Cris, R. , S. Buckmaster , C. Bain , and A. Bonn . 2011. “UK Peatland Restoration—Demonstrating Success.” IUCN UK National Committee Peatland Programme. Edinburgh.

[ppl70325-bib-0122] Cris, R. , S. Buckmaster , C. Bain , and M. Reed . 2014. “Global Peatland Restoration Demonstrating Success.” Edinburgh: IUCN UK National Committee Peatland Programme.

[ppl70325-bib-0123] Daniels, R. E. , and A. Eddy . 1990. “Handbook of European Sphagna. Institute of Terrestrial Ecology.” In Natural Environment Research Council. London: HMSO.

[ppl70325-bib-0019] de Mendiburu, F. 2021. “Agricolae: Statistical Procedures for Agricultural Research. R Package Version 1, 3–5.” https://CRAN.R‐project.org/package=agricolae.

[ppl70325-bib-0020] Dieleman, C. M. , Z. Lindo , J. W. McLaughlin , A. E. Craig , and B. A. Branfireun . 2016. “Climate Change Effects on Peatland Decomposition and Porewater Dissolved Organic Carbon Biogeochemistry.” Biogeochemistry 128: 385–396.

[ppl70325-bib-0021] Dilks, T. J. K. , and M. C. F. Proctor . 1979. “Photosynthesis, Respiration and Water Content in Bryophytes.” New Phytologist 82: 97–114.

[ppl70325-bib-0023] Duckett, J. G. , and S. Pressel . 2022. “Do Moss Sporophytes Maintain Water Balance? New Insights From Sporophyte Water Relations and the Wild Maturation Cycle in *Funaria Hygrometrica* Hedw.” Journal of Bryology 44: 187–198.

[ppl70325-bib-0022] Duckett, J. G. , J. Burch , P. W. Fletcher , et al. 2004. “ *In Vitro* Cultivation of Bryophytes: A Review of Practicalities, Problems, Progress and Promise.” Journal of Bryology 26: 3–20.

[ppl70325-bib-0024] Flanagan, L. B. , and K. H. Syed . 2011. “Stimulation of Both Photosynthesis and Respiration in Response to Warmer and Drier Conditions in a Boreal Peatland Ecosystem.” Global Change Biology 17: 2271–2287.

[ppl70325-bib-0025] Flexas, J. , A. Díaz‐Espejo , J. A. Berry , et al. 2007. “Analysis of Leakage in IRGA'S Leaf Chambers of Open Gas Exchange Systems: Quantification and Its Effects in Photosynthesis Parameterization.” Journal of Experimental Botany 58: 1533–1543. 17339650 10.1093/jxb/erm027

[ppl70325-bib-0026] Fuchs, M. 1990. “Infrared Measurement of Canopy Temperature and Detection of Plant Water Stress.” Theoretical and Applied Climatology 42: 253–261.

[ppl70325-bib-0027] Fukuta, E. , A. Sasaki , and T. Nakatsubo . 2012. “Microclimate and Production of Peat Moss *Sphagnum Palustre* L. in the Warm‐Temperate Zone.” Plant Species Biology 27, no. 1: 110–118.

[ppl70325-bib-0028] Furness, S. B. , and J. P. Grime . 1982a. “Growth Rate and Temperature Responses in Bryophytes: I. An Investigation of *Brachythecium Rutabulum* .” Journal of Ecology 70: 513–523.

[ppl70325-bib-0029] Furness, S. B. , and J. P. Grime . 1982b. “Growth Rate and Temperature Responses in Bryophytes: II. A Comparative Study of Species of Contrasted Ecology.” Journal of Ecology 70: 525–536.

[ppl70325-bib-0030] Gaberščik, A. , and A. Martinčič . 1987. “Seasonal Dynamics of Net Photosynthesis and Productivity of *Sphagnum Papillosum* .” Lindbergia 13: 105–110.

[ppl70325-bib-0031] Gaudig, G. , M. Krebs , A. Prager , et al. 2018. “ *Sphagnum* Farming From Species Selection to the Production of Growing Media: A Review.” Mires and Peat 20: 1–30.

[ppl70325-bib-0032] Gerdol, R. , and R. Vicentini . 2011. “Response to Heat Stress of Populations of Two *Sphagnum* Species From Alpine Bogs at Different Altitudes.” Environmental and Experimental Botany 74: 22–30.

[ppl70325-bib-0033] Glime, J. M. 2007. “Bryophyte Ecology.” In Michigan Technological University, Botanical Society of America & International Association of Bryologists. Chapman and Hall.

[ppl70325-bib-0121] Gong, J. , N. Roulet , S. Frolking , H. Peltola , A. M. Laine , N. Kokkonen , and ES. Tuittila . 2020. “Modelling the Habitat Preference of Two Key *Sphagnum* Species in a Poor Fen as Controlled by Capitulum Water Content.” Biogeosciences 17: 5693–5719.

[ppl70325-bib-0034] Goulden, M. L. , and P. M. Crill . 1997. “Automated Measurements of CO_2_ Exchange at the Moss Surface of a Black Spruce Forest.” Tree Physiology 17: 537–542. 14759826 10.1093/treephys/17.8-9.537

[ppl70325-bib-0035] Grace, J. , and T. C. Marks . 1978. “Physiological Aspects of Bog Production at Moor House.” In Production Ecology of British Moors and Montane Grasslands, edited by D. A. Wardle , J. G. Canadell , S. Díaz , G. Heldmaier , R. B. Jackson , D. F. Levia , E. D. Schulze , and U. Sommer , 38–51. Springer.

[ppl70325-bib-0036] Graham, L. E. , E. Kim , P. Arancibia‐Avila , J. M. Graham , and L. W. Wilcox . 2010. “Evolutionary and Ecophysiological Significance of Sugar Utilization by the Peat Moss *Sphagnum Compactum* (Sphagnaceae) and the Common Charophycean Associates *Cylindrocystis Brebissonii* and *Mougeotia* sp. (Zygnemataceae).” American Journal of Botany 97: 1485–1491. 21616902 10.3732/ajb.0900341

[ppl70325-bib-0037] Griffiths, H. , K. Maxwell , D. Richardson , and W. Robe . 2004. “Turning the Land Green: Inferring Photosynthetic Physiology and Diffusive Limitations in Early Bryophytes.” In The Evolution of Plant Physiology, edited by A. Hemsley and I. Poole , 3–16. Elsevier Academic Press.

[ppl70325-bib-0038] Grime, J. P. , E. R. Rincon , and B. E. Wickerson . 1990. “Bryophytes and Plant Strategy Theory.” Botanical Journal of the Linnean Society 104: 175–186.

[ppl70325-bib-0039] Grobe, A. , B. Tiemeyer , and M. Graf . 2021. “Recommendations for Successful Establishment of Sphagnum Farming on Shallow Highly Decomposed Peat.” Mires and Peat 27: 1–18.

[ppl70325-bib-0040] Gunnarsson, U. 2005. “Global Patterns of *Sphagnum* Productivity.” Journal of Bryology 27: 269–279.

[ppl70325-bib-0041] Günther, A. , G. Jurasinski , K. Albrecht , G. Gaudig , M. Krebs , and S. Glatzel . 2017. “Greenhouse Gas Balance of an Establishing *Sphagnum* Culture on a Former Bog Grassland in Germany.” Mires and Peat 20: 1–16.

[ppl70325-bib-0044] Hájek, T. , and E. Vicherová . 2014. “Desiccation Tolerance of *Sphagnum* Revisited: A Puzzle Resolved.” Plant Biology 16: 765–773. 25068160 10.1111/plb.12126

[ppl70325-bib-0042] Hájek, T. , and R. P. Beckett . 2008. “Effect of Water Content Components on Desiccation and Recovery in *Sphagnum* Mosses.” Annals of Botany 101: 165–173. 18024417 10.1093/aob/mcm287 PMC2701845

[ppl70325-bib-0043] Hájek, T. , E. S. Tuittila , M. Ilomets , and R. Laiho . 2009. “Light Responses of Mire Mosses–a Key to Survival After Water‐Level Drawdown?” Oikos 118: 240–250.

[ppl70325-bib-0045] Hanson, P. J. , N. A. Griffiths , C. M. Iversen , et al. 2020. “Rapid Net Carbon Loss From a Whole‐Ecosystem Warmed Peatland.” AGU Advances 1: e2020AV000163.

[ppl70325-bib-0046] He, X. , K. S. He , and J. Hyvonen . 2016. “Will Bryophytes Survive in a Warming World?” Perspectives in Plant Ecology, Evolution and Systematics 19: 49–60.

[ppl70325-bib-0047] Heskel, M. A. , O. S. O'sullivan , and P. B. Reich . 2016. “Convergence in the Temperature Response of Leaf Respiration Across Biomes and Plant Functional Types.” Proceedings of the National Academy of Sciences of the United States of America 113: 3832–3837. 27001849 10.1073/pnas.1520282113 PMC4833281

[ppl70325-bib-0048] Horsley, K. , L. R. Stark , and D. N. McLetchie . 2011. “Does the Silver Moss *Bryum argenteum* Exhibit Sex‐Specific Patterns in Vegetative Growth Rate, Asexual Fitness or Prezygotic Reproductive Investment?” Annals of Botany 107: 897–907. 21320878 10.1093/aob/mcr027 PMC3080617

[ppl70325-bib-0049] Hoshi, Y. 2017. “ *Sphagnum* Growth in Floating Cultures: Effect of Planting Design.” Mires and Peat 20: 1–10.

[ppl70325-bib-0050] Jauhiainen, J. , and J. Silvola . 1999. “Photosynthesis of *Sphagnum Fuscum* at Long‐Term Raised CO_2_ Concentrations.” Annales Botanici Fennici 36: 11–19.

[ppl70325-bib-0051] Karki, S. , T. P. Kandel , L. Elsgaard , R. Labouriau , and P. E. Lærke . 2019. “Annual CO_2_ Fluxes From a Cultivated Fen With Perennial Grasses During Two Initial Years of Rewetting.” Mires and Peat 25: 1–22.

[ppl70325-bib-0052] Kellner, E. , and S. Halldin . 2002. “Water Budget and Surface‐Layer Water Storage in a *Sphagnum* Bog in Central Sweden.” Hydrological Processes 16: 87–103.

[ppl70325-bib-0053] Kurnianto, S. , M. Warren , J. Talbot , B. Kauffman , D. Murdiyarso , and S. Frolking . 2015. “Carbon Accumulation of Tropical Peatlands Over Millennia: A Modeling Approach.” Global Change Biology 21: 431–444. 25044171 10.1111/gcb.12672

[ppl70325-bib-0054] Laing, C. , G. Granath , L. R. Belyea , K. E. Allton , and H. Rydin . 2014. “Tradeoffs and Scaling of Functional Traits in *Sphagnum* as Drivers of Carbon Cycling in Peatlands.” Oikos 123: 817–828.

[ppl70325-bib-0055] Lembrechts, J. J. , and J. Lenoir . 2020. “Microclimatic Conditions Anywhere at any Time!” Global Change Biology 26: 337–339. 31799715 10.1111/gcb.14942

[ppl70325-bib-0056] Lembrechts, J. J. , J. Lenoir , N. Roth , et al. 2019. “Comparing Temperature Data Sources for Use in Species Distribution Models: From In‐Situ Logging to Remote Sensing.” Global Ecology and Biogeography 28: 1578–1596.

[ppl70325-bib-0057] Li, Y. , X. Song , S. Li , W. T. Salter , and M. M. Barbour . 2020. “The Role of Leaf Water Potential in the Temperature Response of Mesophyll Conductance.” New Phytologist 225: 1193–1205. 31545519 10.1111/nph.16214

[ppl70325-bib-0058] Limpens, J. , F. Berendse , C. Blodau , et al. 2008. “Peatlands and the Carbon Cycle: From Local Processes to Global Implications–a Synthesis.” Biogeosciences 5: 1475–1491.

[ppl70325-bib-0059] Lösch, R. , P. Mülders , E. Fischer , and J. P. Frahm . 1994. “Scientific Results of the BRYOTROP Expedition to Zaire and Rwanda. 3. Photosynthetic Gas Exchange of Bryophytes From Different Forest Types in Eastern Central Africa.” Tropical Bryology 9: 169–185.

[ppl70325-bib-0060] Lunt, P. H. , R. M. Fyfe , and A. D. Tappin . 2019. “Role of Recent Climate Change on Carbon Sequestration in Peatland Systems.” Science of the Total Environment 667: 348–358. 30833238 10.1016/j.scitotenv.2019.02.239

[ppl70325-bib-0061] Ma, X. , H. Xu , Z. Cao , L. Shu , and R. Zhu . 2022. “Will Climate Change Cause the Global Peatland to Expand or Contract? Evidence From the Habitat Shift Pattern of *Sphagnum* Mosses.” Global Change Biology 28: 6419–6432. 35900846 10.1111/gcb.16354

[ppl70325-bib-0062] Márquez, D. A. , and F. A. Busch . 2024. “The Interplay of Short‐Term Mesophyll and Stomatal Conductance Responses Under Variable Environmental Conditions.” Plant, Cell & Environment 47: 3393–3410. 10.1111/pce.14880 38488802

[ppl70325-bib-0063] Marschall, M. , and P. Borbély . 2011. “Photosynthetic Responses of the Desiccation Intolerant *Sphagnum Angustifolium* in Relation to Increasing Its Desiccation Tolerance by Exogenous ABA.” Acta Biologica Szegediensis 55: 119–121.

[ppl70325-bib-0064] Matsuda, T. 1968. “Ecological Study of the Moss Community and Microorganisms in the Vicinity of Syowa Station, Antarctica.” JARE Scientific Reports. Ser. E, Biology 29: 1–58.

[ppl70325-bib-0065] McCarter, C. P. , and J. S. Price . 2014. “Ecohydrology of *Sphagnum* Moss Hummocks: Mechanisms of Capitula Water Supply and Simulated Effects of Evaporation.” Ecohydrology 7: 33–44.

[ppl70325-bib-0066] Melosik, I. , and S. M. Såstad . 2005. “ *In Vitro* Propagation of Selected *Sphagnum* Species (Section Subsecunda).” Lindbergia 30: 21–31.

[ppl70325-bib-0067] Mendoza, V. , M. Pazos , R. Garduño , and B. Mendoza . 2021. “Thermodynamics of Climate Change Between Cloud Cover, Atmospheric Temperature and Humidity.” Scientific Reports 11: 21244. 34711818 10.1038/s41598-021-00555-5 PMC8553890

[ppl70325-bib-0068] Moore, P. A. , T. G. Pypker , and J. M. Waddington . 2013. “Effect of Long‐Term Water Table Manipulation on Peatland Evapotranspiration.” Agricultural and Forest Meteorology 178: 106–119.

[ppl70325-bib-0069] Murray, K. J. , J. D. Tenhunen , and R. S. Nowak . 1993. “Photoinhibition as a Control on Photosynthesis and Production of *Sphagnum* Mosses.” Oecologia 96: 200–207. 28313416 10.1007/BF00317733

[ppl70325-bib-0070] Nakanishi, R. , and S. Tsuyuzaki . 2024. “Litter Decomposition Rates in a Post‐Mined Peatland: Determining Factors Studied in Litterbag Experiments.” Environmental Processes 11, no. 1: 2.

[ppl70325-bib-0071] Oechel, W. C. , and K. Van Cleve . 1986. “The Role of Bryophytes in Nutrient Cycling in the Taiga.” In Forest Ecosystems in the Alaskan Taiga: A Synthesis of Structure and Function, edited by K. Cleve , F. S. Chapin , P. W. Flanagan , L. A. Viereck , and C. T. Dyrness , 121–137. Springer New York.

[ppl70325-bib-0072] Page, S. E. , and A. J. Baird . 2016. “Peatlands and Global Change: Response and Resilience.” Annual Review of Environment and Resources 41: 35–57.

[ppl70325-bib-0073] Pannewitz, S. , T. A. Green , K. Maysek , et al. 2005. “Photosynthetic Responses of Three Common Mosses From Continental Antarctica.” Antarctic Science 17: 341–352.

[ppl70325-bib-0074] Perera‐Castro, A. V. , Á. M. González‐Rodríguez , and B. Fernández‐Marín . 2022. “When Time Is Not of the Essence: Constraints to the Carbon Balance of Bryophytes.” Journal of Experimental Botany 73: 4562–4575. 35298628 10.1093/jxb/erac104

[ppl70325-bib-0075] Perera‐Castro, A. V. , and M. Nadal . 2025. “Data From: Modelling Net CO_2_ Assimilation of Two Sphagnum Species From Temperature and Water Content Response. Zenodo.” 10.5281/zenodo.15463352. PMC12163974 40511634

[ppl70325-bib-0077] Perera‐Castro, A. V. , M. J. Waterman , J. D. Turnbull , et al. 2020. “It Is Hot in the Sun: Antarctic Mosses Have High Temperature Optima for Photosynthesis Despite Cold Climate.” Frontiers in Plant Science 11: 1178. 32922412 10.3389/fpls.2020.01178 PMC7457050

[ppl70325-bib-0076] Perera‐Castro, A. V. , M. J. Waterman , S. A. Robinson , and J. Flexas . 2022. “Limitations to Photosynthesis in Bryophytes: Certainties and Uncertainties Regarding Methodology.” Journal of Experimental Botany 73: 4592–4604. 35524766 10.1093/jxb/erac189

[ppl70325-bib-0078] Piatkowski, B. T. , and A. J. Shaw . 2019. “Functional Trait Evolution in *Sphagnum* Peat Mosses and Its Relationship to Niche Construction.” New Phytologist 223: 939–949. 30924950 10.1111/nph.15825

[ppl70325-bib-0079] Pinheiro, J. , D. Bates , and R Core Team . 2023. “nlme: Linear and Nonlinear Mixed Effects Models. R Package Version 3: 1–162.” https://CRAN.R‐project.org/package=nlme.

[ppl70325-bib-0080] Proctor, M. C. 2000. “The Bryophyte Paradox: Tolerance of Desiccation, Evasion of Drought.” Plant Ecology 151: 41–49.

[ppl70325-bib-0083] Proctor, M. C. F. , and Z. Tuba . 2002. “Poikilohydry and Homoiohydry: Antithesis or Spectrum of Possibilities?” New Phytologist 156: 327–349. 33873572 10.1046/j.1469-8137.2002.00526.x

[ppl70325-bib-0082] Proctor, M. C. F. , M. J. Oliver , A. J. Wood , et al. 2007. “Desiccation‐Tolerance in Bryophytes: A Review.” Bryologist 110: 595–621.

[ppl70325-bib-0081] Proctor, M. C. F. , Z. Nagy , Z. Csintalan , and Z. Takács . 1998. “Water Content Components in Bryophytes: Analysis of Pressure Volume Relationships.” Journal of Experimental Botany 49: 1845–1854.

[ppl70325-bib-0084] Qiu, C. , D. Zhu , P. Ciais , B. Guenet , and S. Peng . 2020. “The Role of Northern Peatlands in the Global Carbon Cycle for the 21st Century.” Global Ecology and Biogeography 29: 956–973.

[ppl70325-bib-0085] R Core Team . 2023. R: A Language and Environment for Statistical Computing. R Foundation for Statistical Computing. https://www.R‐project.org/.

[ppl70325-bib-0086] Raven, J. A. 2002. “Putting the Fight in Bryophytes.” New Phytologist 156: 321–323. 33873583 10.1046/j.1469-8137.2002.00545.x

[ppl70325-bib-0087] Raven, J. A. , and D. Edwards . 2004. “Physiological Evolution of Lower Embryophytes: Adaptations to the Terrestrial Environment.” In The Evolution of Plant Physiology, edited by A. R. Hemsley and I. Poole , 17–41. Elsevier Academic Press.

[ppl70325-bib-0089] Rice, S. K. , N. Neal , J. Mango , and K. Black . 2011. “Modeling Bryophyte Productivity Across Gradients of Water Availability Using Canopy Form Function Relationships.” In Tuba Z, Stack NG, Stark LR, Bryophyte Ecology and Climate Change, 441–457. University Press, Cambridge.

[ppl70325-bib-0088] Rice, S. K. , T. A. Gagliardi , and R. A. Krasa . 2018. “Canopy Structure Affects Temperature Distributions and Free Convection in Moss Shoot Systems.” American Journal of Botany 105: 1499–1511. 30114317 10.1002/ajb2.1145

[ppl70325-bib-0090] Rudolph, H. 1968. “Gas Exchange Determinations of *Sphagnum magellanicum* : A Contribution to the Problem of Membrane Pigments in Sphagna (III).” Planta 79: 35–43. 24522820 10.1007/BF00388819

[ppl70325-bib-0091] Schenker, R. , and W. Block . 1986. “Micro‐Arthropod Activity in Three Contrasting Terrestrial Habitats on Signy Island, Maritime Antarctic.” British Antarctic Survey Bulletin 71: 31–43.

[ppl70325-bib-0092] Schipperges, B. , and H. Rydin . 1998. “Response of Photosynthesis of Sphagnum Species From Contrasting Microhabitats to Tissue Water Content and Repeated Desiccation.” New Phytologist 140: 677–684. 33862962 10.1046/j.1469-8137.1998.00311.x

[ppl70325-bib-0093] Serk, H. , M. B. Nilsson , J. Figueira , T. Wieloch , and J. Schleucher . 2021. “CO_2_ Fertilization of *Sphagnum* Peat Mosses Is Modulated by Water Table Level and Other Environmental Factors.” Plant, Cell & Environment 44: 1756–1768. 10.1111/pce.14043 33751592

[ppl70325-bib-0094] Silvan, N. , K. Jokinen , J. Näkkilä , and R. Tahvonen . 2017. “Swift Recovery of *Sphagnum* Carpet and Carbon Sequestration After Shallow *Sphagnum* Biomass Harvesting.” Mires and Peat 20: 1–11.

[ppl70325-bib-0095] Silvola, J. , and H. Aaltonen . 1984. “Water Content and Photosynthesis in the Peat Mosses *Shagnum Fuscum* and *S. Angustifolium* .” Annales Botanici Fennici 21: 1–6.

[ppl70325-bib-0096] Silvola, J. , and I. Hanski . 1979. “Carbon Accumulation in a Raised Bog. Simulation on the Basis of Laboratory Measurements of CO₂ Exchange.” Oecologia 37: 285–295. 28309216 10.1007/BF00347906

[ppl70325-bib-0097] Silvola, J. , and S. Heikkinen . 1979. “CO_2_ Exchange in the *Empetrum nigrum* ‐ *Sphagnum fuscum* Community.” Oecologia 37: 273–283. 28309215 10.1007/BF00347905

[ppl70325-bib-0098] Sim, T. G. , G. T. Swindles , P. J. Morris , et al. 2021. “Divergent Responses of Permafrost Peatlands to Recent Climate Change.” Environmental Research Letters 16: 034001.

[ppl70325-bib-0099] Smith, E. L. 1936. “Photosynthesis in Relation to Light and Carbon Dioxide.” Proceedings of the National Academy of Sciences of the United States of America 22: 504–511. 16577734 10.1073/pnas.22.8.504 PMC1079215

[ppl70325-bib-0100] Smith, R. I. L. 1996. “Terrestrial and Freshwater Biotic Components of the Western Antarctic Peninsula.” In Foundations for Ecological Research West of the Antarctic Peninsula, edited by R. M. Ross , I. I. E. Hoffmann , and L. B. Quetin , 15–59. American Geophysical Union.

[ppl70325-bib-0101] Stålfelt, M. G. 1937. “Der Gasaustausch der Moose.” Planta 27: 30–60.

[ppl70325-bib-0102] Street, L. E. , P. C. Stoy , M. Sommerkorn , et al. 2012. “Seasonal Bryophyte Productivity in the Sub‐Arctic: A Comparison With Vascular Plants.” Functional Ecology 26: 365–378.

[ppl70325-bib-0103] Swindles, G. T. , J. Holden , C. L. Raby , et al. 2015. “Testing Peatland Water‐Table Depth Transfer Functions Using High‐Resolution Hydrological Monitoring Data.” Quaternary Science Reviews 120: 107–117.

[ppl70325-bib-0104] Tanneberger, F. , C. Tegetmeyer , S. Busse , et al. 2017. “The Peatland Map of Europe.” Mires and Peat 19: 1–17.

[ppl70325-bib-0105] Thompson, D. K. , and J. M. Waddington . 2008. “ *Shagnum* Under Pressure: Towards an Ecohydrological Approach to Examining *Sphagnum* Productivity.” Ecohydrology 1: 299–308.

[ppl70325-bib-0106] Titus, J. E. , and D. J. Wagner . 1984. “Carbon Balance for Two *Sphagnum* Mosses: Water Balance Resolves a Physiological Paradox.” Ecology 65: 1765–1774.

[ppl70325-bib-0107] Updegraff, K. , S. D. Bridgham , J. Pastor , P. Weishampel , and C. Harth . 2001. “Response of CO_2_ and CH_4_ Emissions From Peatlands to Warming and Water Table Manipulation.” Ecological Applications 11: 311–326.

[ppl70325-bib-0108] Van Breemen, N. 1995. “How *Sphagnum* Bogs Down Other Plants.” Trends in Ecology & Evolution 10: 270–275. 21237035 10.1016/0169-5347(95)90007-1

[ppl70325-bib-0109] Velez‐Ramirez, A. I. , W. van Ieperen , D. Vreugdenhil , and F. F. Millenaar . 2011. “Plants Under Continuous Light.” Trends in Plant Science 16: 310–318. 21396878 10.1016/j.tplants.2011.02.003

[ppl70325-bib-0110] Venäläinen, A. , I. Lehtonen , M. Laapas , et al. 2020. “Climate Change Induces Multiple Risks to Boreal Forests and Forestry in Finland: A Literature Review.” Global Change Biology 26: 4178–4196. 32449267 10.1111/gcb.15183 PMC7383623

[ppl70325-bib-0111] Vile, M. A. , K. D. Scott , E. Brault , R. K. Wieder , and D. H. Vitt . 2011. “Living on the Edge: The Effects of Drought on Canada's Western Boreal Peatlands.” In Bryophyte Ecology and Climate Change, edited by Z. Tuba , N. G. Slack , and L. R. Stark , 277–298. Cambridge University Press.

[ppl70325-bib-0112] Vitt, D. H. , B. Crandall‐Stotler , and A. Wood . 2014. “Bryophytes: Survival in a Dry World Through Tolerance and Avoidance.” In Plant Ecology and Evolution in Harsh Environments, edited by N. Rajakaruna , R. Boyd , and T. Harris , 267–295. Nova Publishers.

[ppl70325-bib-0113] Walker, A. P. , K. R. Carter , L. Gu , et al. 2017. “Biophysical Drivers of Seasonal Variability in *Sphagnum* Gross Primary Production in a Northern Temperate Bog.” Journal of Geophysical Research: Biogeosciences 122: 1078–1097.

[ppl70325-bib-0114] Walter, H. 1931. Die Hydratur der Pflanze und ihre physiologisch‐ökologische Bedeutung. Fischer.

[ppl70325-bib-0115] Wang, Z. , and M. Y. Bader . 2018. “Associations Between Shoot‐Level Water Relations and Photosynthetic Responses to Water and Light in 12 Moss Species.” AoB Plants 10: ply034. 29977488 10.1093/aobpla/ply034 PMC6012793

[ppl70325-bib-0116] Wickham, H. 2011. “The Split‐Apply‐Combine Strategy for Data Analysis.” Journal of Statistical Software 40: 1–29.

[ppl70325-bib-0117] Wickham, H. 2016. ggplot2: Elegant Graphics for Data Analysis. Springer‐Verlag.

[ppl70325-bib-0118] Wu, J. , and N. T. Roulet . 2014. “Climate Change Reduces the Capacity of Northern Peatlands to Absorb the Atmospheric Carbon Dioxide: The Different Responses of Bogs and Fens.” Global Biogeochemical Cycles 28: 1005–1024.

[ppl70325-bib-0119] Yin, H. , A. V. Perera‐Castro , K. L. Randall , et al. 2023. “Basking in the Sun: How Mosses Photosynthesise and Survive in Antarctica.” Photosynthesis Research 158: 151–169. 37515652 10.1007/s11120-023-01040-y PMC10684656

[ppl70325-bib-0120] Zhao, B. , and Q. Zhuang . 2023. “Peatlands and Their Carbon Dynamics in Northern High Latitudes From 1990 to 2300: A Process‐Based Biogeochemistry Model Analysis.” Biogeosciences 20: 251–270.

