# Supplementary material for: Modelling Net CO2 Assimilation of Two Sphagnum Species From Temperature and Water Content Response
Source: Physiol Plant. 2025 Jun 13;177(3):e70325. doi: 10.1111/ppl.70325 (PMC12163974; doi:10.1111/ppl.70325)
Supplement: Supplementary file 1 — Figure S1. Custom‐made moss cuvette for moss placement during gas exchange measurement. Figure S2. Linear regression between the CO2 sample factor and the water vapour difference between sample and reference IRGAs. Figure S3. Relationship between moss canopy temperature (T moss) of the two used sensors: infra‐red sensor and thermocouple. Figure S4. Relationship between PAR measured at the beginning of the cycle of moss temperature measurements and at the end of each cycle. Figure S5. Air temperature and photosynthetic active radiation from 15 May to 15 September 2023 of SMEAR II Hyytiälä forest meteorological station. Figure S6. Net CO2 assimilation under light conditions and dark respiration in response to water content during a dehydration curve at 25°C. Figure S7. Response of maximum yield of PSII to water content of samples during air‐dehydration. Table S1. Fitted parameters of the A N Model (Equations (3), (4), (5)) for estimating net CO2 assimilation rates of S. angustifolium and S. squarrosum . Table S2. Fitted parameters of the Model 2 (Equation 6) for estimating moss canopy temperature from PAR, water content and air temperature. [file PPL-177-e70325-s001.pdf]

SUPLEMENTARY INFORMATION

Modelling net CO_2 assimilation of two *Sphagnum* species from temperature and water content response

Alicia V. Perera-Castro, Miquel Nadal

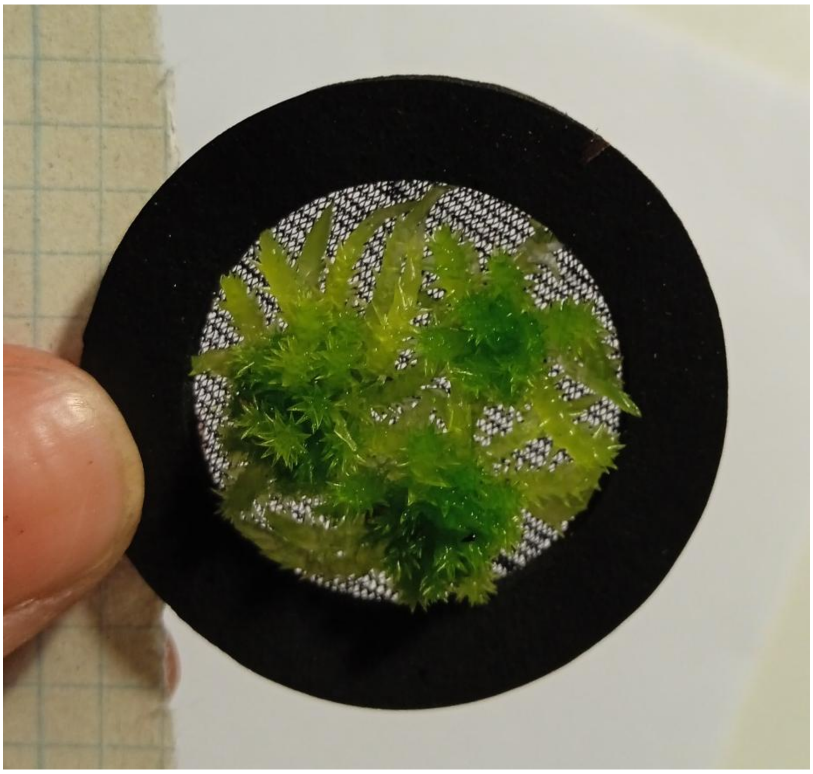

Figure S1. Custom-made moss cuvette consisting of a LI-6800 6 cm^2 gasket affixed to a piece of thin polyester stocking fabric. Normally 3 apical green capitula (top, living part of the moss) of *Sphagnum angustifolium* and *S. squarrosum* were placed on the fabric with shoot overlap minimized. The moss cuvette (with sample over it) were placed inside Li-6800 chamber between the two gaskets of its articulated aperture, creating a kind of “sandwich” of gaskets when the chamber was closed. Both net CO_2 assimilation and dark respiration were based to the projected area of the capitula.

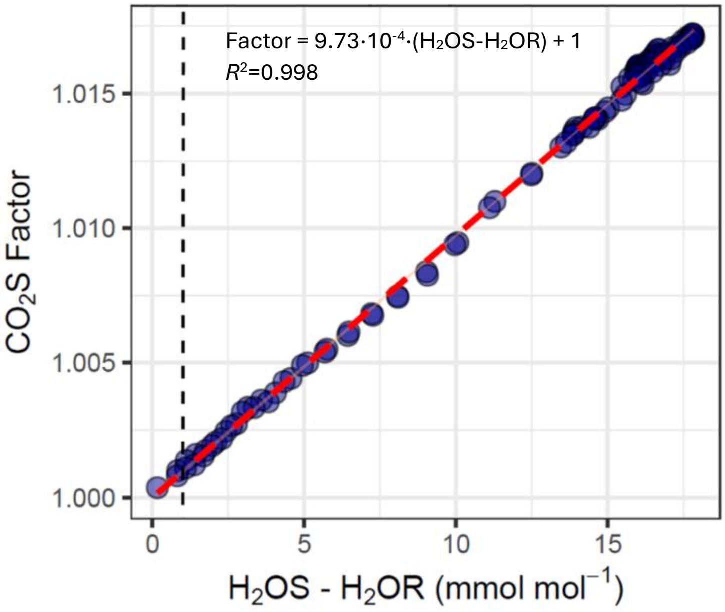

Figure S2. Linear regression between the CO_2 sample (CO_2S) factor and the water vapour difference between sample and reference IRGAs (H_2OS and H_2OS, respectively). The CO_2S factor was calculated as the ratio of CO_2 reference and sample (CO_2R/CO_2S). Data were collected during the dehydration of a wet piece of paper inside the empty moss cuvette.

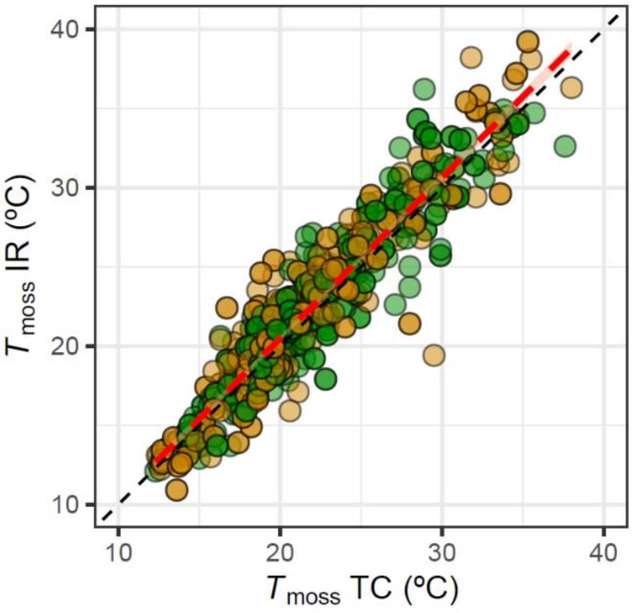

Figure S3. Relationship between moss canopy temperature (T_{moss}) of the two used sensors: infra-red sensor (IR) and thermocouple (TC) for *S. angustifolium* (brown) and *S. squarrosum* (green). Both temperature values were averaged for the final analysis.

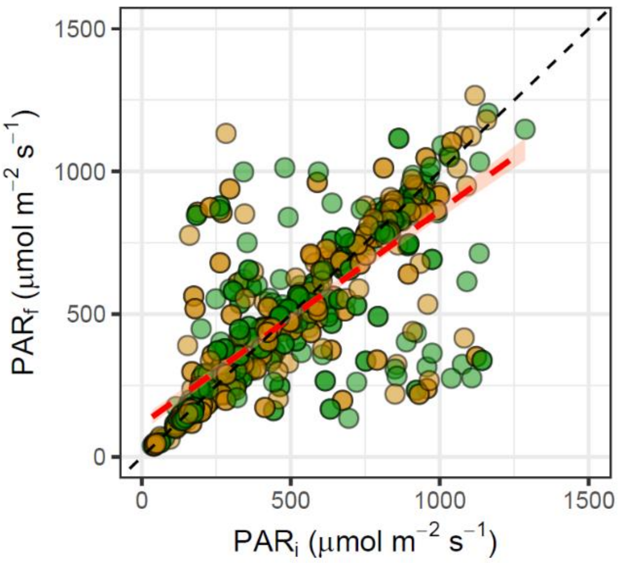

Figure S4. Relationship between PAR measured at the beginning of the cycle of moss temperature measurements (PAR_i) and at the end of each cycle (PAR_f). Some differences were found for those data that were collected in a sunny-cloud transition. Both PAR values were averaged for the final analysis.

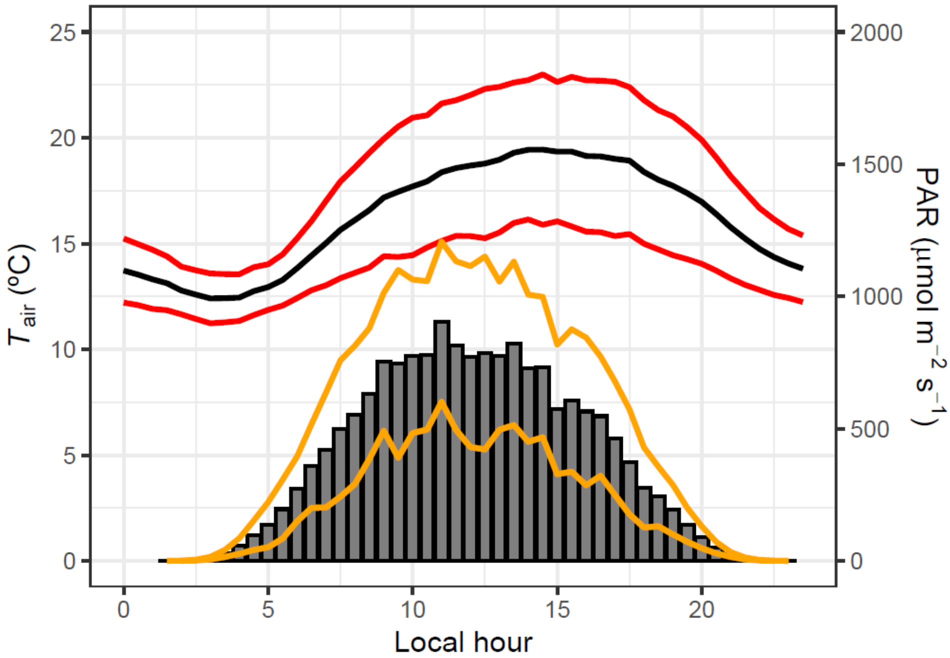

Figure S5. Averaged daily variation of air temperature (T_{air}) and photosynthetic active radiation (PAR) from 3rd to 17th September 2023 of SMEAR II Hyytiälä forest meteorological station. Red line indicates standard deviation of T_{air}. Bars indicate daily average PAR ± standard deviation (orange lines) measured with a Li-Cor Li-190SZ quantum sensor.

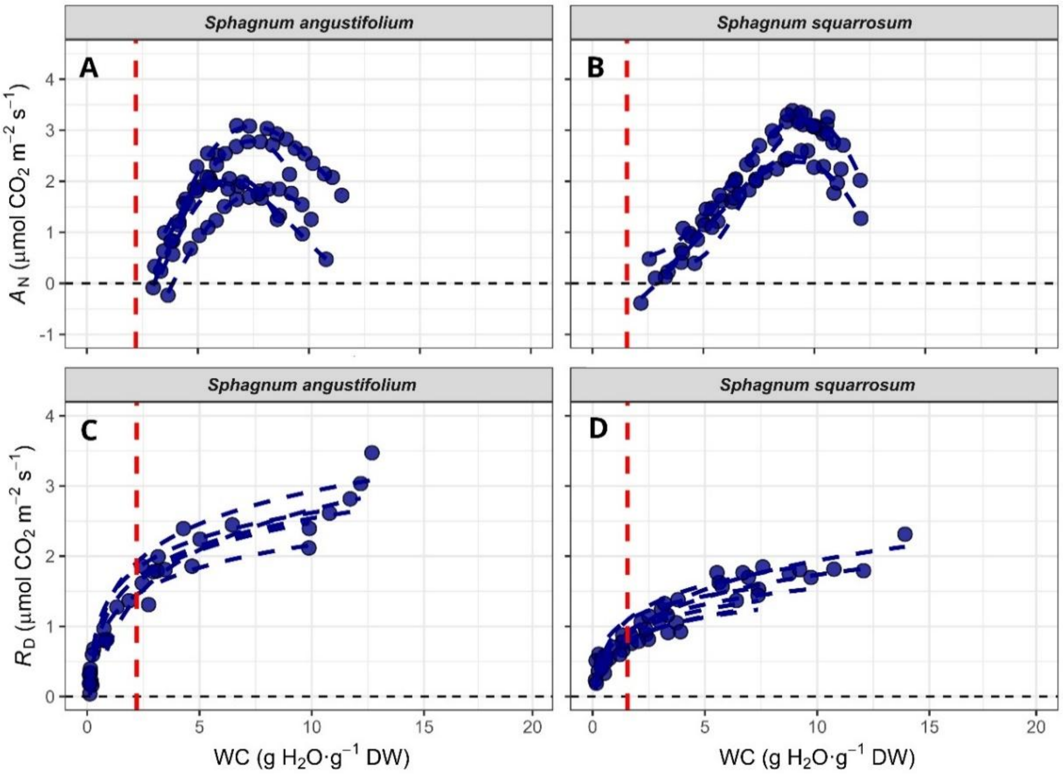

Figure S6. Net CO_2 assimilation (A_N, A, B) under saturated light conditions and dark respiration rates (R_D, C, D) in response to water content (WC) of *S. angustifolium* (A, C) and *S. squarrosum* (B, D) during dehydration curves at 25ºC. Red dashed line indicates the water content at which F_v/F_m was not able to recover to 50% of its original value after rehydration (WC_{FvFm50} = 1.5 and 2.7 g H_2O g^{-1} DW for *S. squarrosum* and *S. angustifolium*, respectively) (Supplementary Fig. S7). Blue dashed lines indicate polynomial and logarithmic fitting for A_N and R_D curves, respectively (n = 5).

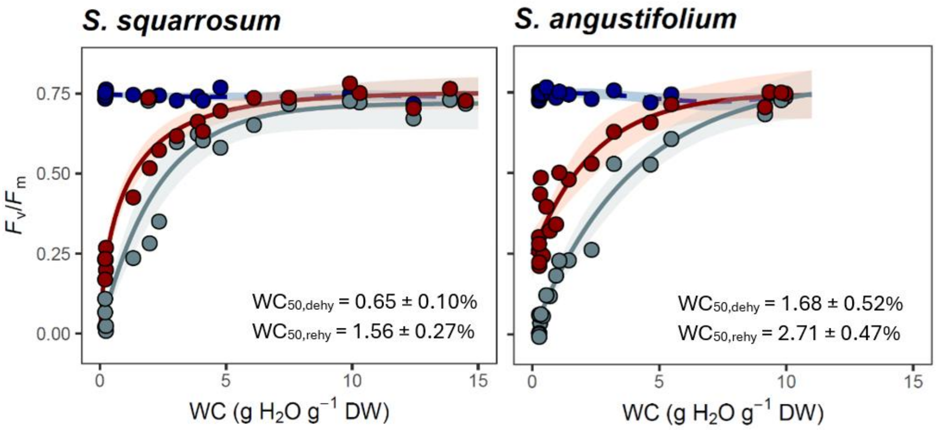

Figure S7. Response of maximum yield of PSII (F_v/F_m) to water content of samples during air-dehydration (red dots) for *S. squarrosum* and *S. angustifolium*. Values of F_v/F_m of the same sample under well-water conditions previous to dehydration (blue dots) and after dehydration + recovery by covering the sample for 24 h with a wet tissue (grey dots) are also represented in the graphs. The water content at which F_v/F_m is reduced by 50 % during dehydration and rehydration ($WC_{50,dehy}$ and $WC_{50,rehy}$, respectively) is shown for both species.

Table S1. Fitted parameters of the A_N Model (eqn 3, 4 and 5) for estimating net CO_2 assimilation rates of *S. angustifolium* and *S. squarrosum*.

Parameter	***Sphagnum angustifolium***		***Sphagnum squarrosum***	
	**MEAN**	**SD**	**MEAN**	**SD**
a	-0.0081	0.0013***	-0.0040	0.0008***
b	0.4490	0.0676***	0.2366	0.0407***
c	-9.4755	0.8508***	-7.0975	0.4904***
d	0.0886	0.0056***	0.0800	0.0057***
e	-1.4964	0.0803***	-1.4275	0.0135***
f	-2.1442	0.0618***	-2.5836	0.0576***
g	-0.0478	0.0128***	-0.0744	0.0097***
h	-0.0602	0.0915	-0.1108	0.0599*
i	0.0222	0.0012***	0.0114	0.0009***

***P<0.001; **P<0.01; *P<0.05

Table S2. Fitted parameters of the Model 2 for estimating moss canopy temperature of both *S. angustifolium* and *S. squarrosum* (merged dataset) from PAR, water content and air temperature of the meteorological station SMEAR II Hyytiälä forest, Finland.

Parameter	MEAN	SD
j	$-8.268\cdot10^{-6}$	$1.358\cdot10^{-6}$***
k	0.0178	0.0014***
l	-0.7343	0.3585*
m	-1.624	0.0687***

***P<0.001; **P<0.01; *P<0.05
